# Extracellular vesicles from mesenchymal stem cells reduce neuroinflammation in hippocampus and restore cognitive function in hyperammonemic rats

**DOI:** 10.1186/s12974-022-02688-4

**Published:** 2023-01-02

**Authors:** Paula Izquierdo-Altarejos, Andrea Cabrera-Pastor, Mar Martínez-García, Carlos Sánchez-Huertas, Alberto Hernández, Victoria Moreno-Manzano, Vicente Felipo

**Affiliations:** 1 Laboratory of Neurobiology, Centro Investigación Príncipe Felipe, Eduardo Primo-Yufera 3, 46012 Valencia, Spain; 2 Fundación Investigación Hospital Clínico, Instituto de Investigación Sanitaria, INCLIVA, Valencia, Spain; 3 Neuronal and Tissue Regeneration Laboratory, Centro Investigación Príncipe Felipe, Valencia, Spain; 4 Laboratory of Bilateral Neural Circuits, Instituto de Neurociencias (CSIC-UMH), Alicante, Spain; 5 Optical and Confocal Microscopy Service, Centro Investigación Príncipe Felipe, Valencia, Spain

**Keywords:** Hyperammonemia, Neuroinflammation, Cognitive impairment, Extracellular vesicles, Mesenchymal stem cells

## Abstract

Chronic hyperammonemia, a main contributor to hepatic encephalopathy (HE), leads to neuroinflammation which alters neurotransmission leading to cognitive impairment. There are no specific treatments for the neurological alterations in HE. Extracellular vesicles (EVs) from mesenchymal stem cells (MSCs) reduce neuroinflammation in some pathological conditions. The aims were to assess if treatment of hyperammonemic rats with EVs from MSCs restores cognitive function and analyze the underlying mechanisms. EVs injected in vivo reach the hippocampus and restore performance of hyperammonemic rats in object location, object recognition, short-term memory in the Y-maze and reference memory in the radial maze. Hyperammonemic rats show reduced TGFβ levels and membrane expression of TGFβ receptors in hippocampus. This leads to microglia activation and reduced Smad7–IkB pathway, which induces NF-κB nuclear translocation in neurons, increasing IL-1β which alters AMPA and NMDA receptors membrane expression, leading to cognitive impairment. These effects are reversed by TGFβ in the EVs from MSCs, which activates TGFβ receptors, reducing microglia activation and NF-κB nuclear translocation in neurons by normalizing the Smad7–IkB pathway. This normalizes IL-1β, AMPA and NMDA receptors membrane expression and, therefore, cognitive function. EVs from MSCs may be useful to improve cognitive function in patients with hyperammonemia and minimal HE.

## Background

Patients with liver cirrhosis may show hepatic encephalopathy (HE), a complex neuropsychiatric syndrome which may progress to coma and death. Many patients with cirrhosis who do not show evident symptoms of HE show minimal HE (MHE), with attention deficits, mild cognitive impairment, psychomotor slowing and motor in-coordination. MHE is associated with reduced performance in daily tasks, quality of life and life span and increased risk of accidents, falls, hospitalizations and clinical HE. MHE is an important health, social and economic problem [27]. However, there are no specific treatments for the neurological alterations of MHE.

Hyperammonemia is a main contributor to the cognitive and motor alterations of MHE [30, 72, 73], which are reproduced by animal models of chronic hyperammonemia [15, 41, 44, 57, 66, 79, 80]. Studies in rats with chronic hyperammonemia and MHE show that the cognitive and motor alterations are a consequence of altered neurotransmission which, in turn, is a consequence of neuroinflammation (reviewed in Cabrera-Pastor et al. [14]). For example, hyperammonemia induces neuroinflammation in hippocampus, with activation of microglia and astrocytes and increased pro-inflammatory factors such as IL-1β and TNFα. This neuroinflammation alters membrane expression of NMDA and AMPA receptors leading to impairment of spatial learning and memory [14, 15, 40, 57, 78, 80]. These alterations may be reversed by reducing neuroinflammation and this can be done by increasing cGMP, with anti-inflammatories or modulating GABAergic neurotransmission [14, 15, 40, 57, 78, 80]. However, these treatments may have secondary effects in patients with liver cirrhosis and other procedures to reduce neuroinflammation would have more therapeutic utility.

Recent reports show that extracellular vesicles (EVs) from mesenchymal stem cells (MSCs) reduce neuroinflammation in different pathological situations. MSCs are multipotent non-hematopoietic cells that present immunomodulatory, anti-inflammatory and regenerative properties [31, 77, 82]. They exert their therapeutic effects mainly in a paracrine manner, secreting different chemokines, cytokines and growth factors [25, 33, 88, 92]. EVs released by MSCs can recapitulate the beneficial effects of the parental MSCs, emerging as a promising alternative to whole cell therapy [6, 55]. EVs released by MSCs act as mediators between the MSCs and the target cells, carrying bioactive proteins, microRNAs and lipids [38]. The use of EVs derived from MSCs presents several advantages for clinical use compared to whole cell therapy including higher safety profile and lower immunogenic capacity [54].

MSC-derived EVs therapy emerges as a promising strategy to treat diseases with an inflammatory component, such as inflammatory bowel disease, arthritis, sepsis, graft-versus-host disease, multiple sclerosis and type I diabetes [3, 35, 46, 47, 49, 61, 95].

Riazifar et al. [65] showed that EVs derived from MSCs activated with IFNγ reduce neuroinflammation and demyelination and improve functional outcomes in a chronic experimental autoimmune encephalomyelitis (EAE) murine model.

Reza-Zaldivar et al. [64] observed that MSC-derived EVs enhance neurogenesis and restore cognitive function in a mouse model of Alzheimer’s disease established by injection of beta amyloid 1−42 aggregates into dentate gyrus.

In stroke, intravenous administration of MSCs-derived EVs increases neurogenesis, neurite remodeling, and angiogenesis, improving animals’ functional recovery [90]. Similar results were observed in a traumatic brain injury model, showing an inflammation reduction and improved outcomes after MSCs-derived EVs administration [96]. Injection of MSCs-derived exosomes has also been proved to reduce inflammation and promote neuro-regeneration in a rat model of spinal cord injury [24, 37].

On the basis of these findings, we hypothesized that injecting EVs from MSCs to hyperammonemic rats could reduce neuroinflammation and restore cognitive function. The initial aims of this work were to assess whether injection of EVs from MSCs to hyperammonemic rats: (1) reduces neuroinflammation, activation of microglia and astrocytes in hippocampus; and (2) restores different forms of learning and memory modulated by hippocampus (short-term memory, object location, object recognition, working memory and reference memory).

Rats were made hyperammonemic by feeding them an ammonia-containing diet. Once the rats were hyperammonemic they were injected with EVs from adipose tissue MSCs and the effects on neuroinflammation and cognitive function were analyzed. These studies showed that EVs from MSCs reduce neuroinflammation, including microglia and astrocytes activation in hippocampus and restore cognitive function. A third aim of this study was to advance in the understanding of the mechanisms involved in the beneficial effects of EVs on neuroinflammation and cognitive function. To reach this aim we developed a system using freshly isolated hippocampal slices from control and hyperammonemic rats treated ex vivo with EVs from MSCs. This system reproduced the EVs-induced reduction of microglia and astrocytes activation and of neuroinflammation in hippocampus of hyperammonemic rats and was used to analyze the underlying mechanisms.

## Materials and methods

### Study design, model of chronic hyperammonemia and in vivo treatment with MSC-EVs

Male Wistar rats (Charles River Laboratories, Barcelona, Spain) were made hyperammonemic by feeding them an ammonium-containing diet as in [4, 29, 78]. The diet contained ammonium acetate (25% by weight). In this model of chronic moderate hyperammonemia ammonia concentration in blood increases nearly threefold and in brain by around 50% [4]. These increases are similar to those found in patients with liver cirrhosis [28].

Animals were distributed into four groups (*n* = 18 rats per group): control rats injected with PBS, phosphate-buffered saline, (C + PBS); control rats treated with MSC-derived extracellular vesicles (C + EVs); hyperammonemic rats injected with PBS (HA + PBS) and hyperammonemic rats treated with MSC-EVs (HA + EVs).

**Fig. 1:**
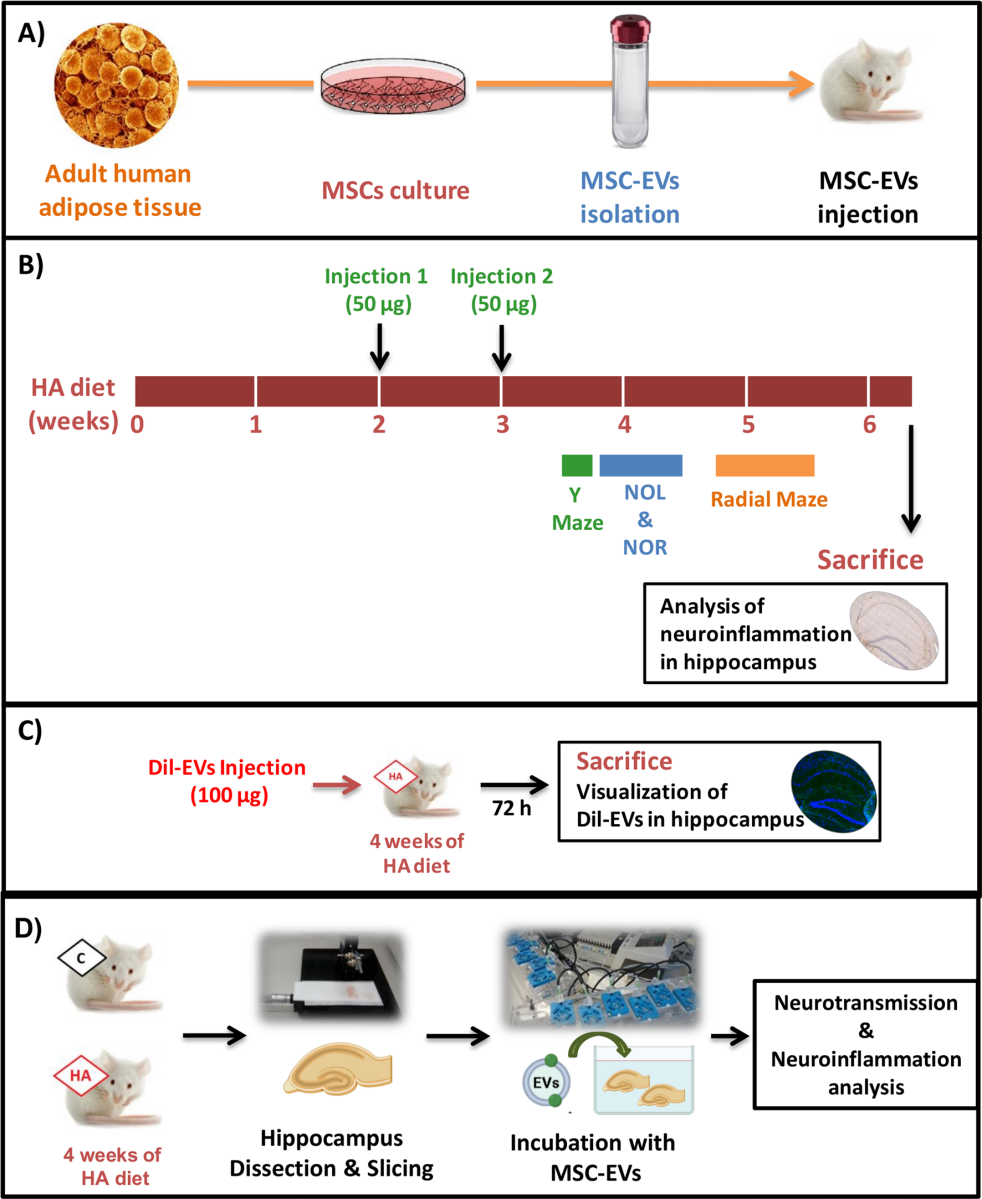
Study design. **A** Human adipocyte derived mesenchymal stem cells (MSCs) were cultured and extracellular vesicles were isolated from the culture media. **B** After 2 weeks of starting the hyperammonemic diet, HA and control rats were intravenously injected in the tail vein either with 50 µg of protein of isolated vesicles from MSCs or PBS as vehicle. A second injection was performed one week later. Behavioral tests (Y-maze, novel object location, novel object recognition and 8-radial maze) were performed 10–20 days after first injection to assess cognitive function. Rats were killed during week 6 of hyperammonemia to extract the brain for neuroinflammation analysis. **C** We injected fluorescently labeled EVs into different rats with 4 weeks of HA (*n* = 2). Rats were killed after 3 days and hippocampi were extracted to assess whether fluorescent EVs reach this area. **D** Ex vivo experiments were performed to investigate the molecular pathways involved: control and HA rats were killed and the hippocampi were dissected and sliced. Hippocampal slices from HA rats were incubated with EVs derived from MSCs during 30 min. Control and HA slices without EVs incubation were included as reference. Additional pre-treatments of the MSC-EVs and controls were included (see “Materials and methods” section). After the incubation, slices were processed for neurotransmission and neuroinflammation analysis

The experimental design is summarized in Fig. 1. After 2 weeks of hyperammonemia, rats were intravenously injected in the tail vein either with 50 µg of protein of EVs from MSCs in 300 μL (equivalent to 1.25 × 10^10^ vesicles), or the same volume of PBS as vehicle. A second injection was performed 1 week later. This dosing paradigm was selected because we used the same one in a previous study [42] showing that injection of EVs from hyperammonemic rats to normal rats induces neuroinflammation in cerebellum and motor in-coordination. This suggests that a similar paradigm could be useful to test the effects of MSC-derived EVs and was also within the dosing range of MSC-EVs reported in the literature. Behavioral tests were performed 10–20 days after first injection and rats were killed at day 25 to extract the brain for further analyses as described below. All the experiments were approved by the Comite de Experimentación y Bienestar Animal (CEBA) of our Center and by Conselleria de Agricultura of Generalitat Valenciana and were performed in accordance with guidelines of the Directive of the European Commission (2010/63/EU) for care and management of experimental animals.

### MSC culture and EVs isolation

Human adipocyte-derived mesenchymal stem cells, kindly given by HistoCell Ltd. (Spain), obtained from subcutaneous fat as previously described [16] were used for EVs isolation. MSCs were obtained from subcutaneous fat from lipoaspirates of five different female donors. Cells were characterized by Histocell [16] according to International Society of Cell Therapy (ISCT) minimum criteria for adipose-derived stromal and stem cells. Immunophenotypic analyses by flow cytometry revealed positive expression (> 95%) of CD13, CD44, CD73, CD90 and CD105, and negative expression (< 2%) of CD14, CD19, CD34, CD45 and HLA-DR. The adherent culture of MSCs exhibited the expected fibroblast-like spindle-shaped morphology. Cells also showed multipotency capacity to differentiate towards adipogenic, chondrogenic and osteogenic lineages, as confirmed by corresponding differentiation assays. For EVs isolation, MSCs were used at passage 4 to 6. Cells were expanded and grown in growth medium (GM: high glucose DMEM basal medium supplemented with 20% FBS (previously centrifuged at 100,000*g* for 1 h and then filtered through 0.2-um filter for EVs depletion), 100 units/mL penicillin and 100 µg/mL streptomycin and 2 mM l-glutamine). Each culture and isolation round consisted of 15 plates (diameter: 150 mm) at a seeding density of 750,000 cells/plate. Sub-confluent cells were incubated in GM for 48 h and then media were collected and cleared from detached cells and cells fragments by centrifugation at 300×*g* and then, the supernatant at 2000×*g* for 10 min, respectively. Subsequently, apoptotic bodies and other cellular debris were pelleted by centrifugation of the resulting supernatant at 10,000×*g* for 30 min. EVs were then pelleted from the previous resulting supernatant at 100,000×*g* for 1 h. The EV pellets were washed with PBS and centrifuged at 100,000×*g* for 1 h. The EVs were finally suspended into 100 μL PBS. Protein content was measured using the Pierce BCA-200 Protein Assay Kit (ThermoFisher, Grand Island, NY, USA) according to the manufacturer's instructions and samples were stored at – 80 °C.

### Methods for hTGFβ1-shRNA design, cloning and lentivirus production

For lentivirus production, the target sequences to deplete human TGFβ1 (TGFβ#1: 5′-GCAGCTGTACATTGACTTT; TGFβ#2: 5′-CAAGCAGAGTACACACAGCAT) were cloned for expression as shRNA into the pLL3.7 vector (plasmid #11795; Addgene), which encoded GFP in a separate locus. To generate this constructs, sense and antisense oligonucleotides were annealed and ligated into the HpaI/XhoI sites of the pLL3.7 vector. The pLL3.7 plasmid encoding luciferase shRNA (5′-CTTACGCTGAGTACTTCGA-3′) was kindly provided by Lüders. Lentivirus were generated using the LentiLox3.7 system [67]. Briefly, HEK293T cells were cotransfected with the pLL3.7 constructs and the packaging plasmids. Lentiviral particles in the medium were concentrated by centrifugation at 26,000 rpm during 2 h, resuspended in chilled PBS, aliquoted and stored at – 80 °C. Viral titers were obtained by infecting HEK293T cells with serial dilutions of concentrated lentiviruses and sorting of GFP-positive cells by FACS 72 h after infection. For exosomes purification, HEK293T cells were infected at a multiplicity of infection (moi) of 5. The complete medium was replaced with fresh medium 16–18 h after infection. The infection efficiencies were higher than 70% for shRNA-TGFβ1, shRNA-TGFβ#2 and shRNA-luciferase, respectively, determined by the number of cells expressing GFP. The knockdown expression efficiency, evaluated by western blotting using the TGFβ antibody (Abcam), was higher than 60% over the total protein expression either in 293T cells after plasmid transfection, and in hADSC after lentiviral infection.

### Transmission electron microscopy

Isolated vesicles were observed in negative staining mode, using a copper grid covered by a “holey film” carbon layer and the contrast staining was performed with a uranyl acetate solution 1% w/v. Grids were viewed using a FEI Tecnai G2 Spirit (FEI Europe, Eindhoven, Netherlands) and photographed with Olympus digital camera (Soft Image Solutions GmbH, Germany).

### Nanoparticle tracking analysis

Distribution profile, size and quantity of vesicles were assessed by Nanoparticle Tracking Analysis with a NanoSight NS300 system (Malvern, UK). A 1:1000 dilution of EVs samples was used for NTA. 5 videos of 30 s were recorded at random points of each sample and were analyzed with NTA 3.2 Dev Build 3.2.16 software.

### Biomarker characterization of EVs by immunoblotting

Samples were subjected to electrophoresis and immunoblotting as described in [29]. Primary antibodies used to characterize the EVs were against Alix (1:1000, Proteintech), Hsp70 (1:1000, Proteintech), Flotillin-2 (1:500, Fisher), CD9 (1:1000, Abcam), TGFβ (1:1000, Abcam) and β-actin (1:5000, Abcam) as loading control. We also evaluated the presence/absence of positive and negative EV markers in EVs and non-EV fractions (whole cell lysates, supernatant discarded after the last ultracentrifugation step (SP) and cell culture medium (CCM)) using the following primary antibodies as negative markers for EVs: Calnexin (1:1000, Novus Biologicals), Lamin A/C (1:500, Santa Cruz) and Histone3 (1:1000, Abcam). Secondary antibodies were anti-rabbit or anti-mouse IgG conjugated with alkaline phosphatase (1:4000, Sigma) except for CD9. For CD9 we used a secondary antibody conjugated to HRP-peroxidase (1:2500, Sigma) and chemiluminescent signal was obtained adding the SuperSignal West Femto Maximum Sensitivity Substrate (Thermo Scientific) according to the manufacturer instructions. CD9 images were acquired using an Alliance Q9 Advanced (Uvitec).

### Fluorescent labeling of extracellular vesicles

To check whether injected EVs reach the brain, we performed a parallel experiment in different rats (*n* = 2 per group). During the isolation protocol, EVs were labeled with the lipophilic dye Dil (Sigma) by incubating the pellets of the first ultracentrifugation with 40 μg/mL of dye during 15 min. Control and hyperammonemic rats were intravenously injected with 100 μg of fluorescently labeled EVs and 72 h later were anesthetized with sodium pentobarbital and transcardially perfused with 0.9% saline followed by 4% paraformaldehyde in 0.1 M phosphate buffer (pH 7.4). Fixed brains were extracted and frozen in OCT. 10 µm sections were cut on a cryostat and counterstained with Iba1 (1:300, WAKO), NeuN (1:200, Millipore), GFAP (1:400, Sigma) and Alix (1:200, Proteintech), followed by goat anti-rabbit or goat anti-mouse Alexa 488 secondary antibody (1:400, Invitrogen) and DAPI staining. The antibody against Alix recognizes both human and rat Alix. Images were acquired with a Leica TCS SP8 (Leica Microsystems Heidelberg GmbH, Mannheim, Germany) inverted laser scanning confocal microscope using oil objectives: 63X Plan-Apochromat-Lambda Blue 1.4 N.A. This experiment aimed to assess if EVs injected i.v. reach or not the hippocampus and which cell types incorporate the EVs. We used a single injection of 100 μg of fluorescently labeled EVs instead of the two injections of 50 μg used for therapeutic treatment of the rats because these EVs were fluorescently labeled and the fluorescence must be later detected by confocal microscopy. To ensure that the intensity of fluorescence is high enough to visualize the EVs we used a larger dose of EVs. A similar biodistribution was found in control and hyperammonemic rats.

### Evaluation of spatial learning in the 8-arm radial maze

Rats were kept under a caloric restricted diet during the test to maintain the motivation to seek for food. On the first day, rats were habituated to the maze (one session of 5 min with reward pellets disseminated on the entire maze, followed by one session of 5 min with pellets in the end of the arms). The test was performed in the following 4 days with 5 trials per day. In each trial, the rat was placed in the center of the maze with reward food in four of the arms. Configurations of food location were specific for each rat and were kept invariable through the test. The trial ended after the rat found all the pellets or after a maximum of 3 min. The number of reference memory errors (unbaited arms visited) and working memory errors (entries to arms already visited in the same trial) were calculated. Learning index was defined as the difference between the number of right choices and reference errors as in Hernández-Rabaza et al. [40].

### Evaluation of short-term spatial recognition memory in the Y-maze

This test is based on the rodents’ innate curiosity to explore novel areas and presents no negative or positive reinforcement and very little stress for the rats. The protocol is a modification of the test used by Sarnyai et al. [69] and Sanderson et al. [68]. The rat was placed into the start arm and allowed to explore the maze with one of the arms closed for 2 min (training trial) for three times, with 1 min of inter-trial interval. After that, the rat was placed again in the start arm and allowed to explore freely all three arms of the maze for 2 min (test trial). Time spent in each arm was recorded and the discrimination ratio was calculated as: [(time spent in the novel arm—time spent in the familiar arm)/total time in the two arms] were registered.

### Evaluation of novel object recognition (NOR) and novel object location (NOL) memory

NOR and NOL memory tests were performed as in Taoro-Gonzalez et al. [80] in an open-field arena (70 × 70 × 40 cm) of black painted wood with visuospatial cues on the walls. Rats were habituated during 3 days in 2 sessions of 5 min per day, allowing them to explore the empty arena. NOL test was performed on day 4. It consists of a sample phase and a test phase. During the sample phase, 2 identical objects were place in the cage and the rat was allowed to explore them for 3 min. After a time interval of 2 h, one of the objects was moved to a different location and the rat was allowed to explore the cage again for 3 min. NOR test was performed on day 5. During the sample phase 2 identical objects were place in the cage and the rat was allowed to explore them for 3 min. Test phase was performed after 6 h, with the objects located in the same position but exchanging one of the objects for an unexplored one and allowing the rat to freely explore again for 3 min. Sessions were recorded with a digital camera and the time exploring the familiar stimulus and the novel stimulus (object with different location in the case of NOL and unexplored object in the case of NOR) was counted. Discrimination ratio for each test was calculated as: [(time exploring novel stimulus—time exploring familiar stimulus)/total exploration time].

### Ex vivo experiment: design and treatments

Control and hyperammonemic rats after 4–5 weeks of hyperammonemia were used for the ex vivo experiment. Animals were killed by decapitation and the brain was extracted. The hippocampi were dissected and immersed immediately into ice-cold Krebs buffer (NaCl 119 mM, NaHCO_3_ 26.2 mM, glucose 11 mM, KCl 2.5 mN, CaCl_2_ 2.5 mM, KH_2_PO_4_ 1 mM aerated with 95% O_2_ and 5% CO_2_ at pH 7.4). After that, hippocampi were placed longitudinally on a manual chopper and cut to obtain transverse slices (400 μm). Slices were transferred to incubation wells in a perfusion system (Campden Instruments, Model 7450) and incubated for 15 min at 35.5 °C in Krebs buffer for stabilization. Once stabilized, the slices from hyperammonemic rats were incubated during 30 min at 35.5 °C with the following treatments, all of them dissolved in Krebs buffer and aerated: 1 ng/mL of recombinant TGFβ (Miltenyi Biotec) (HA + rec TGFβ) [62], 1.2 µg/mL of anti-TGFβ antibody (Abcam) (HA + T), 10 µg/mL of extracellular vesicles from MSCs (HA + EVs) [43], 10 µg/mL of extracellular vesicles from MSCs previously treated with 1.2 µg/mL of anti-TGFβ antibody (Abcam) for 1 h at 37 °C (HA + EVs + anti-TGFβ) [83], 10 µg/mL of extracellular vesicles from MSCs lacking TGFβ as described above (HA + EVs lacking TGFβ; 10 µg/mL of extracellular vesicles from MSCs plus 2 µg/mL of galunisertib, an antagonist of TGFβ receptor (HA + EVs + anti-TGFβR [10, 56]. All treatments were for 30 min. We have previously shown that this time is enough to induce the effects on glial activation and on the underlying mechanisms [2, 12, 79]. Slices from control and hyperammonemic rats incubated in Krebs buffer without treatment were included as reference (C and HA, respectively).

### Analysis of neuroinflammation and alterations in neurotransmission in hippocampus

#### Analysis of protein content in hippocampus by western blot and ELISA

Injected animals were killed by decapitation 25 days after first injection and the hippocampi were dissected and homogenized. Hippocampal slices from the ex vivo experiments were collected after the incubation with the treatments and homogenized by sonication for 20 s in a buffer (Tris–HCl 66 mM pH 7.4, SDS 1%, EGTA 1 mM, glycerol 10%, leupeptin 0.2 mg/mL, NaF 1 mM, Na ortho-vanadate 1 mM). Samples were subjected to electrophoresis and immunoblotting as above. Primary antibodies used were against IL-6 (1:500, Invitrogen), IL-1β (1:500, RD Systems), IL-4 (1:1.000, Abcam), IL-10 (1:1.000, Abcam), Arginase1 (1:1.000, Santa Cruz Biotechnology), TNFα (1:500 RD Systems), TGFβ (1:1.000, Abcam), TGFβR1 (1:1.000, Sigma), Smad7 (1:1.000, Invitrogen), Smad2/3 (1:1.000 Cell Signaling), phospho-Smad2/3 (1:1.000, Cell Signaling), IkBα (1:10.000, Abcam), phospho-IkBα (1:10.000, Sigma). β-actin (1:5.000, Abcam) or GAPDH (1:5.000, Millipore) were used as protein loading control. Secondary antibodies were anti-rabbit or anti-mouse IgG conjugated with alkaline phosphatase (1:4.000, Sigma; except for loading controls, were dilution at 1:10,000 was used). Membranes were scanned using the ScanJet 5300C (Hewlett-Packard, Amsterdam, the Netherlands) and band intensities were quantified using Alpha Imager 2200 version 3.1.3 (Alpha Innotech Corporation, San Francisco).

Levels of IL-1β and TNFα in hippocampus homogenates were also determined by ELISA using kits specific for rat (eBioscience, USA), according to manufacturer instructions. Briefly, 20 µL of homogenates obtained as above were diluted fivefold with the kit diluent buffer and added to the 96-well plate. Samples were incubated overnight at 4 °C, washed and incubated for 1 h at room temperature with biotin conjugate, followed by 45-min incubation with streptavidin-HRP solution. Then, the plate was incubated for 30 min in the dark with TMB substrate. Finally, stop solution was added and the optical absorbance was measured at 450 nm with a microplate reader. Concentrations of IL-1β and TNFα were calculated according to the instruction manual and referred to the total protein concentration of each sample.

#### Analysis of microglial and astrocytic activation by immunohistochemistry

Twenty-five days after first injection, four rats of each group were anesthetized with sodium pentobarbital and transcardially perfused with 0.9% saline followed by 4% paraformaldehyde in 0.1 M phosphate buffer (pH 7.4). Brains were removed and post-fixed in the same fixative solution for 24 h at 4 °C. For the ex vivo approach, slices were fixed by immersion in 4% paraformaldehyde in 0.1 M phosphate buffer (pH 7.4) at 4 °C. Paraffin-embedded sections (5 μm) were cut and mounted on coated slide glass. Sections were sequentially incubated with 3% H_2_O_2_ for 15 min to quench endogenous peroxidase activity, blocking serum (normal goat serum or horse serum) and primary antibodies (4ºC, overnight): Iba1 (1:300, Wako) of GFAP (1:400, Sigma), IL-1β (1:200, Abcam), TNFα (1:200, Abcam). Then, slides were incubated with biotinylated secondary antibodies (1:200, Vector Laboratories) goat anti-mouse, goat anti-rabbit and horse anti-goat for 1 h, followed by incubation with VECTASTAIN ABC kit (Vector Laboratories) for 30 min and diaminobenzidine for 10 min. Sections were counterstained with Mayer’s hematoxylin (DAKO) for 5 min. Sections were scanned with an Aperio Versa system (Leica Biosystems, Germany). From these scans, fields at 40× magnification containing the CA1 region were captured using the software ImageScope64. 8–10 images per rat were taken, generally from three different sections of the hippocampus. Microglial activation was analyzed by measuring the area of Iba1-stained cells with IpWin 32 software program and astrocytic activation was analyzed by measuring the GFAP stained area with ImageJ software.

Intensity of IL1β and TNFα in CA1 region was quantified using the ROI manager function in ImageJ: CA1 region was manually selected and four squared regions outside the neuron layer were also taken and considered as background. Inverted values of mean gray value were recorded and the mean intensity of the four background regions was subtracted to the CA1 region intensity. Mean intensity of the different field captured for each rat was calculated and results were expressed as a percentage of control group. IL1β and TNFα were analyzed in the CA1 region because we have previously shown that hyperammonemic rats show increased levels of them in this region [9, 80].

#### Analysis of NF-κB activation

NF-κB activation was measured in the paraffin-embedded sections from the ex vivo experiment. For double immunofluorescence, primary antibodies were NF-κB p50 (1:200, Abcam) and Iba1 (1:300, Abcam), followed by donkey anti-mouse Alexa 488 and donkey anti-rabbit Alexa 647 secondary antibodies (1:400, Invitrogen) and DAPI. Eight images per rat were acquired with a confocal microscope using oil objectives: 63X Plan-Apochromat-Lambda Blue 1.4N.A. Z-stack images were acquired to validate the nuclear localization of p50. The ratio of nuclear/cytoplasmic NF-κB p50 intensity in CA1 region was calculated using ImageJ as previously described in Dadsetan et al. [22]: nuclei in CA1 region were manually outlined using ROI manager function on DAPI blue channel and the selection was applied on green channel (p50) to measure nuclear fluorescence, measuring mean gray value intensity for each nucleus. Pyramidal layer of CA1 region was also outlined and the green fluorescence was measured in this area. Cytoplasmic content of p50 was calculated as: [total green fluorescence in CA1] − [green fluorescence in CA1 nuclei]. Then, cytoplasmic/nuclear ratio of p50 intensity was calculated and values were expressed as percentage of the control group. Number of microglial cells expressing NF-κB was manually counted.

Transcriptional activity of NF-κB p65 subunit was also analyzed in nuclear extracts using a commercial kit (Cayman Chemical, USA) according to manufacturer instructions. Nuclear extracts were prepared from fresh hippocampal slices as follows: slices were homogenized in hypotonic buffer (HEPES 10 mM, KCl 10 mM, EDTA 1 mM, EGTA 1 mM, DTT 1 mM, β-glycerophosphate 10 mM and protease inhibitors) with a Dounce homogenizer. Then, Igepal CA-630 at 0.4% was added and the lysates were centrifuged at 12,000×g for 5 min at 4 °C. Supernatant was discarded and the pellet was sonicated in hypertonic buffer (Tris 10 mM, NaCl 400 mM, Igepal CA-630 0.5%, EDTA 1 mM, EGTA 1 mM, DTT 1 mM, β-glycerophosphate 10 mM and protease inhibitors), left on ice for 30 min and centrifuged at 12,000×*g* for 5 min at 4 °C. The supernatant was collected and used as nuclear extract.

30 µL of nuclear extracts was used to measure p65 transcriptional activity. Samples were added to a 96-well plate coated with a consensus dsDNA sequence that specifically binds p65 transcription factor and incubated overnight at 4 °C. After washing, p65 primary antibody was added and the plate was incubated for 1 h at room temperature, followed by goat anti-rabbit HRP Conjugate incubation for 1 h at room temperature. Then, the plate was incubated for 30 min in the dark with developing solution. Finally, stop solution was added and the optical absorbance was measured at 450 nm with a microplate reader. Data were expressed as percentage of control group.

#### Analysis of membrane expression of receptors by cross-linking with BS3

Membrane expression of the GluA1 and GluA2 subunits of AMPA receptors, NR2B subunit of NMDA receptors and TGFβ receptor 2 were analyzed in hippocampal slices by cross-linking with BS3 (bis(sulfosuccinimidyl) suberate, Rockford). After the treatments (see above), slices were added to tubes containing ice-cold Krebs buffer with or without 2 mM BS3 and incubated for 30 min at 4 °C with gentle shacking. Cross-linking was terminated by quenching the reaction with 100 mM glycine (10 min, 4 °C). The slices were transferred to tubes with buffer (Tris–HCl 66 mM pH 7.4, SDS 1%, EGTA 1 mM, glycerol 10%, leupeptin 0.2 mg/mL, NaF 1 mM, Na ortho-vanadate 1 mM) and homogenized by sonication for 20 s. Samples treated or not with BS3 were analyzed by western blot as describe above, using the following primary antibodies: GluA1 (1:1.000, Millipore), GluA2 (1:2.000, Millipore), NR2B (1:1.000, Millipore), TGFβR2 (1:1.000, Sigma). Secondary antibody was anti-rabbit IgG conjugated with alkaline phosphatase (1:4.000, Sigma). The membrane expression was calculated as the difference between the intensity of the bands without BS3 (total protein) and with BS3 (non-membrane protein), using Alpha Imager 2200 version 3.1.3 (Alpha Innotech Corporation, San Francisco) to quantify the band intensities. Concerning the cell types expressing the receptors analyzed, AMPA and NMDA receptors are mainly expressed in the synaptic membrane of neurons, although they can also be expressed by glial cells [18, 26]. TGFbR2 is detected in almost every cell type in the CNS including neurons, astrocytes, microglia and endothelial cells [23].

### Statistical analysis

Data are expressed as mean ± SEM. All statistical analyses were performed using GraphPad Prism software 8.1.2 version. Data were analyzed by one-way or two-way analysis of variance (ANOVA) followed by Tukey post hoc test. Most of the analyses were performed using one-way ANOVA, except for three parameters of radial maze: evolution of learning index, number of reference memory errors and number or working memory errors along the different days of the test, in which two-way ANOVA was used. A confidence level of 95% was accepted as significant.

## Results

### Characterization of EVs from MSCs and of its transport to hippocampus

**Fig. 2:**
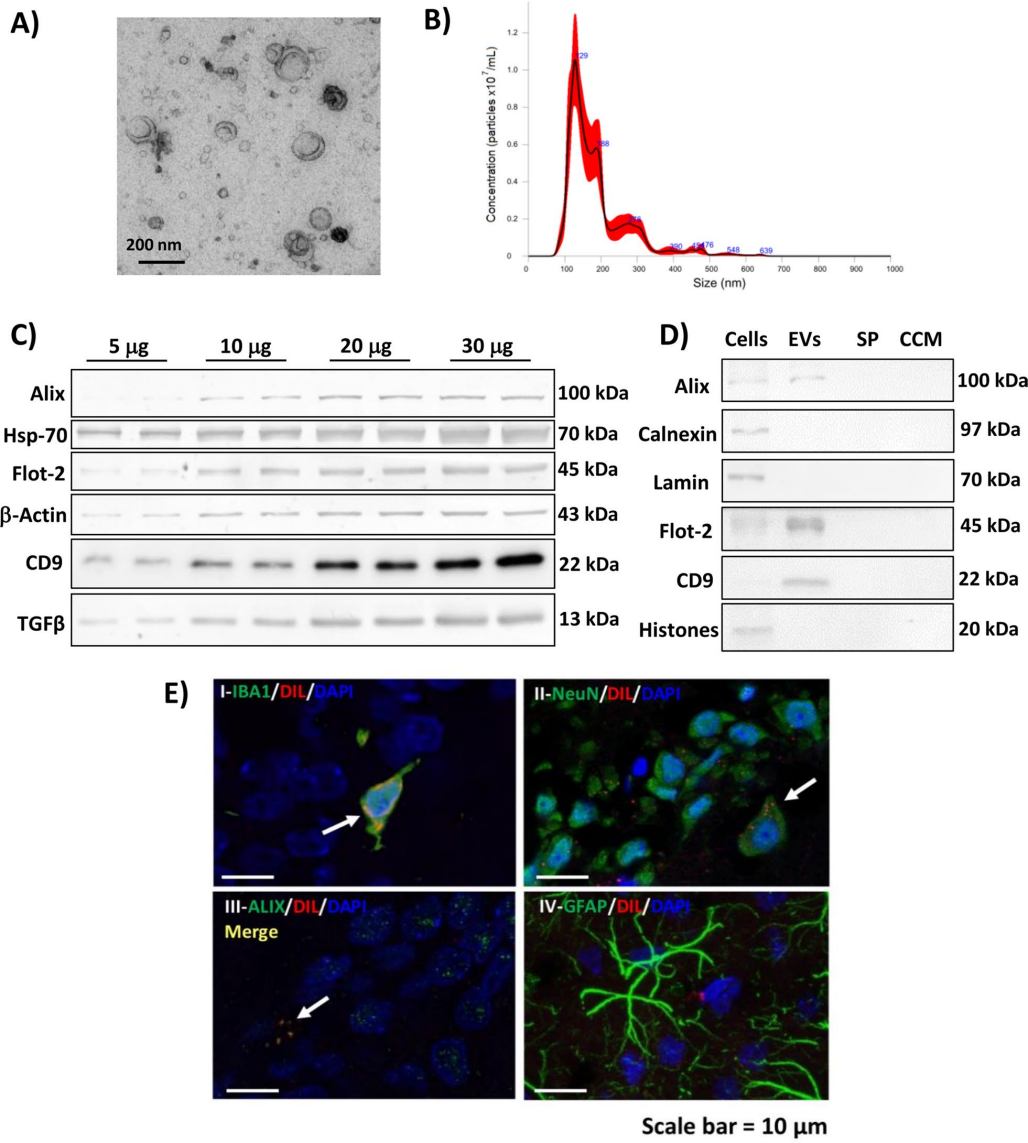
Characterization of extracellular vesicles isolated from human adipocyte derived mesenchymal stem cells. **A** Representative image of EVs obtained by transmission electron microscopy after negative staining. **B** Representative size profile of EVs obtained by Nanoparticle tracking analysis. **C** Representative image of EV markers (Alix, Hsp70, Flotillin-2, CD9), β-actin and TGFβ measured by western blot with different quantities of initial protein. **D** Western blot bands of EV markers (Alix, Flotillin-2 and CD9) and non-EV markers (calnexin, lamin and histones) in origin cell lysates, EVs, supernatant discarded in the last ultracentrifugation step of the EVs isolation procedure (SP) and cell culture medium (CCM). **E** Intravenously injected Dil-labeled extracellular vesicles (red) reach the hippocampus of HA rats after 72 h. Co-localization was found with I microglia and II neurons in the pyramidal layer. III Red fluorescence signal co-localizes with Alix, a marker of extracellular vesicles. IV No clear co-localization was found with astrocytes. Scale bar = 10 µm

Negative staining of the isolated vesicles and visualization by transmission electron microscopy confirmed the presence of small concave-shaped extracellular vesicles (< 200 nm) in the samples (Fig. 2A). Mode size diameter of EVs was 126 ± 8 nm measured by nanoparticle tracking analysis in three replicates, with a concentration of 1.14 ± 0.09 × 10^11^ particles/mL. Representative size profile distribution is shown in Fig. 2B. Western blot analysis shows that EVs isolated from MSCs contain the EV markers Alix, Hsp70, Flotillin-2 and CD9, as well as TGFβ (Fig. 2C). These data confirm that the samples contain true EVs. We also evaluated the presence/absence of positive and negative EV markers in EVs and non-EV fractions (whole cell lysates, supernatant discarded after the last ultracentrifugation step (SP) and cell culture medium (CCM). Isolated EVs were enriched in EV markers such as Alix, Flotillin-2 and CD9, while they lack non-EV markers, such as calnexin, lamin or histones, present in cells (Fig. 2D). As expected, these markers were not detected in the discarded supernatant or the cell culture medium.

Injected EVs reached the hippocampus (Fig. 2E). Dil-labeled EVs (red signal) were detected mainly in microglia (Fig. 2E-I) and pyramidal layer neurons (Fig. 2E-II). The red fluorescence signal of Dil-labeled EVs co-localized with Alix (green fluorescence), a marker of EVs, confirming that it corresponds to injected labeled EVs (Fig. 2E-III). The immunofluorescence against Alix also stained in green some EVs that are not stained in red, indicating that it also stains endogenous EVs. We did not observe a clear co-localization with astrocytes (Fig. 2E-IV). These results are consistent with previous studies conducted by Li et al. [52] and Otero-Ortega et al. [63]. In the study conducted by Li et al. [52] fluorescent EVs were intravenously injected to mice and they observed that the EVs reached the brain and were taken up mainly by microglia (86.8%) and neurons (12.6%) and only in a small percentage (0.8%) by astrocytes. While the level of astrocytic EVs uptake was low, the observation of astrocytic activation was clear, so that the authors speculate that the astrocytic activation was a secondary even of the activated microglia, as reported by Liddelow et al. [53]. Otero-Ortega et al. [63] obtained similar results after intravenous injection of Dil-labeled MSC-EVs from adipose tissue, finding co-localization of the EVs with microglia and neurons in the brain.

### In vivo administration of EVs from MSCs reverse microglial and astrocytic activation in hippocampus of hyperammonemic rats and normalizes TNFα and IL-1β content

**Fig. 3:**
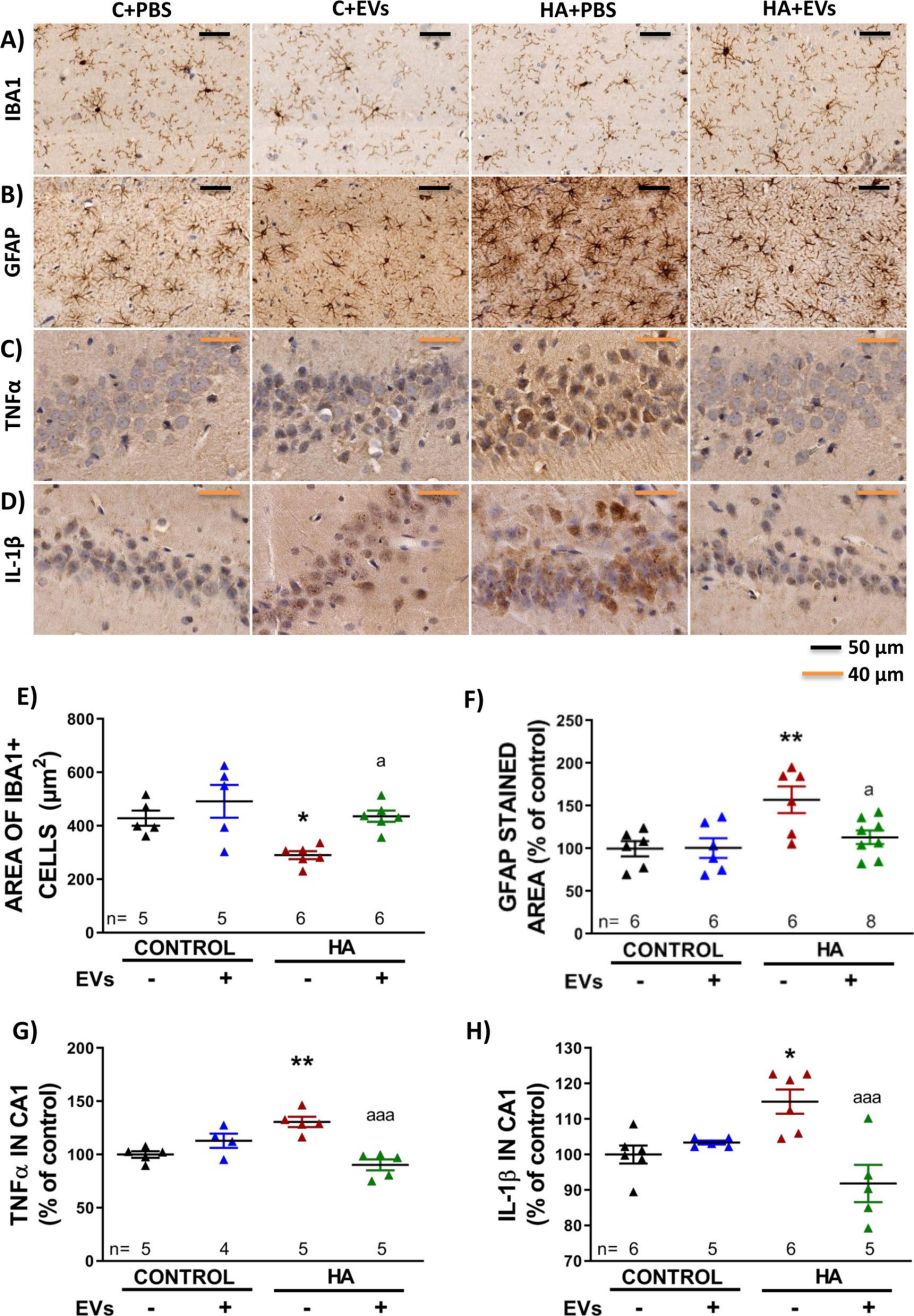
Injected EVs reverse microglial and astrocytic activation and expression of pro-inflammatory markers TNFα and IL-1β in hippocampus. Representative images of **A** immunohistochemistry against Iba-1 and **B** GFAP in hippocampus and **C** TNFα and **D** IL-1β in CA1 region. **E** Area of Iba1-stained cells (*n* = 5–6) and **F** percentage of area stained with GFAP (*n* = 6–8) in hippocampus. **G** Content of TNFα (*n* = 4–5) and **H** IL-1β (*n* = 5–6) in CA1 region of hippocampus, expressed as percentage of controls. One-way ANOVA with Tukey post hoc test was performed to compare all groups. Values are the mean ± SEM. Values significantly different from controls are indicated by asterisk (*p < 0.05; **p < 0.01) and values significantly different between HA + PBS and HA + EVs groups are indicated by a (a = *p* < 0.05; aaa = *p* < 0.001). Sample size of each group is indicated at the bottom of the bars

Hyperammonemic rats show neuroinflammation, with activation of microglia and astrocytes in hippocampus. Activated microglial cells acquire an amoeboid shape and reduce their processes, thus presenting a reduction in their area. The area of microglial cells was reduced in hippocampus of hyperammonemic rats (290 ± 15 μm^2^ versus 428 ± 28 μm^2^ in control rats, *p* < 0.05) and the injection of MSC-EVs reversed this effect (436 ± 21 μm^2^ in comparison with 290 ± 15 μm^2^, *p* < 0.05) (Fig. 3A and E). The percentage of area stained with GFAP increased in hippocampus of hyperammonemic rats (157 ± 15% of control group, *p* < 0.01), reflecting an increase in astrocyte activation (Fig. 3B and F), which was also reversed by injection of MSC-EVs (113 ± 8% of control group, *p* < 0.05). The content of the pro-inflammatory cytokines TNFα and IL-1β were increased in neurons of the CA1 region of hippocampus of hyperammonemic rats (131 ± 5%, *p* < 0.01; and 115 ± 3%, *p* < 0.05, respectively), as shown in Fig. 3C, D, G and H. Injection of MSC-EVs normalized the amount of both cytokines (90 ± 5%, *p* < 0.001; and 92 ± 5%, *p* < 0.001, respectively).

None of these parameters was significantly altered in control rats injected with MSC-EVs compared to control rats injected with PBS (area of microglia: 492 ± 51 μm^2^; percentage of area stained with GFAP in comparison with control group: 101 ± 11%; TNFα content in CA1: 113 ± 7% and IL-1β content in CA1: 103 ± 1%).

### In vivo injection of EVs induce a shift in hippocampus of hyperammonemic rats from a pro-inflammatory to an anti-inflammatory state

**Fig. 4:**
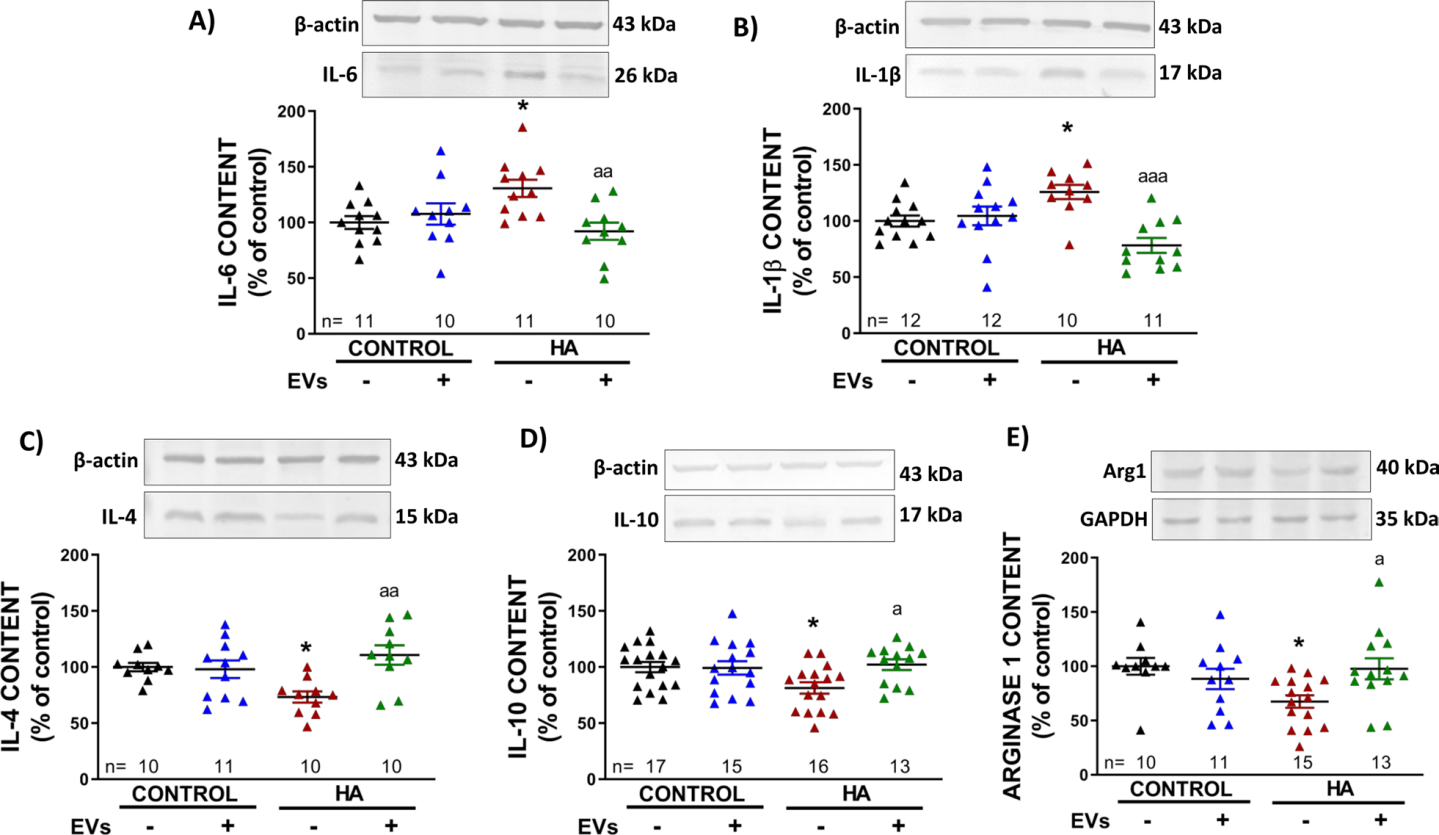
Analysis of neuroinflammation in hippocampus of injected rats analyzed by Western blot. Content of **A** IL-6 (*n* = 10–11), **B** IL-1β (*n* = 10–12), **C** IL-4 (*n* = 10–11), **D** IL-10 (*n* = 13–17) and **E** Arginase1 (*n* = 10–15) in hippocampi homogenates. Representative images of the blots of each protein and the load control (β-actin or GAPDH for Arginase1) are shown. One-way ANOVA with Tukey post hoc test was performed to compare all groups. Values are expressed as percentage of protein content in PBS-injected control rats and are the mean ± SEM. Values significantly different from controls are indicated by asterisk (*p < 0.05) and values significantly different between HA + PBS and HA + EVs groups are indicated by a (a = *p* < 0.05; aa = *p* < 0.01; aaa = *p* < 0.001). Sample size of each group is indicated at the bottom of the bars

The content of pro-inflammatory cytokines IL-6 and IL-1β was increased (131 ± 10%, *p* < 0.05; and 126 ± 6%, *p* < 0.05, respectively) in hippocampi of hyperammonemic rats compared to control rats (Fig. 4A and B). The injection of MSC-EVs normalized the levels of both cytokines (98 ± 8%, *p* < 0.01; and 78 ± 7%, *p* < 0.001, respectively).

In contrast, the amount of anti-inflammatory cytokines IL-4 and IL-10 was reduced (73 ± 5%, *p* < 0.05; and 81 ± 5%, *p* < 0.05) in hyperammonemic rats (Fig. 4C and D) and the injection of MSC-EVs reversed this effect, normalizing IL-4 and IL-10 content (111 ± 9%, *p* < 0.01; and 102 ± 5%, *p* < 0.05, respectively). The content of arginase 1, a marker of anti-inflammatory microglia, was reduced (67 ± 6%, *p* < 0.05) in hippocampi of hyperammonemic rats (Fig. 4E) and was also normalized by injection of MSC-EVs (98 ± 10%, *p* < 0.05 compared to hyperammonemic rats).

None of these parameters was significantly altered in control rats injected with MSC-EVs compared to control rats injected with PBS (IL-6: 108 ± 10%; IL-1β: 105 ± 8%; IL-4: 98 ± 8%; IL-10: 99 ± 6%; Arg1: 88 ± 9%).

### EVs from MSCs restore memory and learning in hyperammonemic rats

**Fig. 5:**
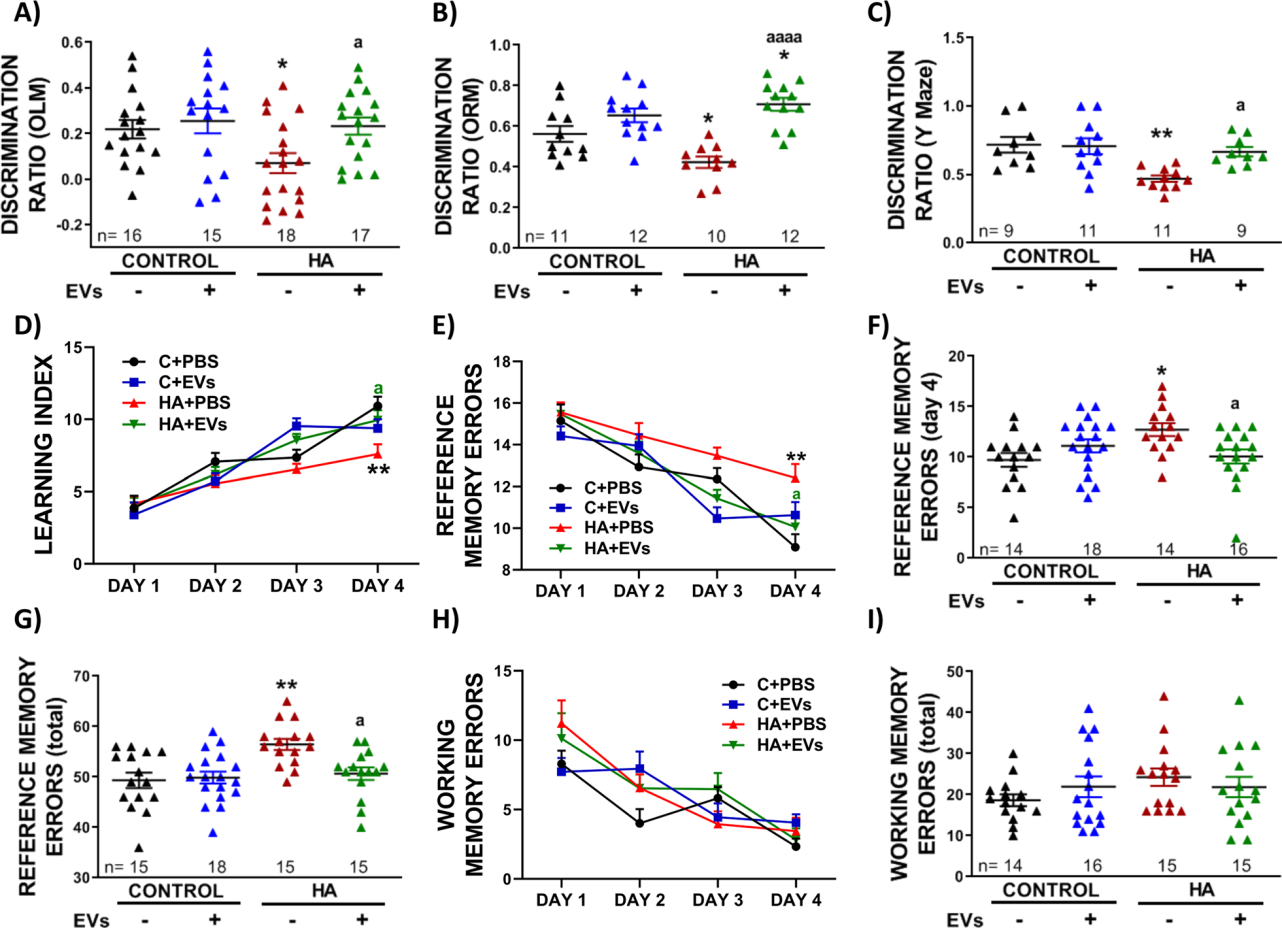
Injection of MSC-EVs restores memory and learning impairments found in HA rats. Discrimination ratio in **A** novel object location (*n* = 15–18), **B** novel object recognition (*n* = 10–12) and **C** Y-maze test (*n* = 9–11). The following panels correspond to radial maze (*n* = 14–18): evolution of **D** learning index and **E** number of reference memory errors, **F** number of reference memory errors at day 4 of the test, **G** total number of reference memory errors, **H** evolution of working memory errors and **I** total number of working memory errors. Values are the mean ± SEM. For sections **A**–**C**, **F**, **G** and **I** one-way ANOVA with Tukey post hoc test was performed to compare all groups. Values significantly different from control group are indicated by asterisk (*p < 0.05; **p < 0.01) and values significantly different between HA + PBS and HA + EVs groups are indicated by a (a = *p* < 0.05; aaaa = *p* < 0.0001). For sections **D**, **E** and **H** two-way ANOVA with Tukey post hoc test was performed to compare all groups. Values significantly different from control group are indicated by asterisk (**p < 0.01) and values significantly different between HA + PBS and HA + EVs groups are indicated by a (a = *p* < 0.05). Sample size of each group is indicated at the bottom of the bars

Hyperammonemic rats showed impaired cognitive function, with reduced discrimination ratio both in object location (0.07 ± 0.04 versus 0.22 ± 0.04 in control rats, *p* < 0.05) and object recognition memory (0.42 ± 0.03 versus 0.56 ± 0.04 in control rats, *p* < 0.05) tests (Fig. 5A and B). Injection of MSC-EVs to hyperammonemic rats reversed this impairment (0.23 ± 0.03 in comparison to 0.07 ± 0.04 in HA rats injected with PBS, *p* < 0.05; and 0.71 ± 0.03 in comparison to 0.42 ± 0.03 in HA rats injected with PBS, *p* < 0.0001, respectively). Hyperammonemic rats injected with EVs showed a discrimination ratio in the OLM similar to control rats and even better than control rats in ORM (Fig. 5A and B). Control rats injected with MSC-EVs showed discrimination ratios similar to control rats injected with PBS in both tests (0.26 ± 0.05 versus 0.22 ± 0.04 in the OLM and 0.65 ± 0.03 versus 0.56 ± 0.04 in the ORM).

Hyperammonemic rats also showed impaired short-term memory as measured by discrimination ratio in Y-maze (0.47 ± 0.02 in comparison to 0.72 ± 0.06 in control rats, *p* < 0.01), which was also reversed by injection of EVs (0.67 ± 0.03 in comparison to 0.47 ± 0.02 in HA rats injected with PBS, *p* < 0.05) (Fig. 5C). Injection of MSC-EVs to control rats does not induce significant differences in this parameter (0.71 ± 0.06 versus 0.72 ± 0.06 in control rats injected with PBS).

Learning, reference memory and working memory were assessed in the 8-arm radial maze. Learning index was significantly lower (7.6 ± 0.7 versus 11 ± 0.6, *p* < 0.01) in hyperammonemic than in control rats at day 4 of the test and was normalized by injection of EVs (10 ± 0.7, *p* < 0.05) (Fig. 5D). Hyperammonemic rats showed impaired reference memory, with increased (13 ± 0.6 versus 10 ± 0.7 in the control group, *p* < 0.05) reference memory errors at day 4 of the test (Fig. 5E and F) and total number of reference memory errors (56 ± 1 versus 49 ± 2 in control rats, *p* < 0.01) (Fig. 5G). Both parameters were normalized by injection of MSC-EVs (10 ± 0.7 in comparison to 13 ± 0.6, *p* < 0.05; and 51 ± 1 in comparison to 56 ± 1, *p* < 0.05). No significant differences were found in working memory errors among experimental groups (Fig. 5H and I), although a tendency towards an increased total number of working memory errors in hyperammonemic rats (24 ± 2 versus 19 ± 1 in control rats) and a certain reduction by the injection of MSC-EVs (22 ± 2 versus 24 ± 2 in HA rats injected with PBS) can be observed in Fig. 5I.

No significant effects were observed in the control rats injected with MSC-EVs in any of the afore-mentioned parameters (learning index at day 4: 9.4 ± 0.6; reference memory errors at day 4: 11 ± 0.7; total reference memory errors: 50 ± 1; and total working memory errors: 22 ± 3).

It should be noted that the beneficial effects of EVs on hyperammonemic rats is not due to reduction of hyperammonemia. Injection of EVs did not affect blood ammonia levels, which were similar in hyperammonemic rats injected (68 ± 6 μM) or not (73 ± 8 μM) with EVs. These levels were higher (*p* < 0.01) than in control rats injected (38 ± 5 μM) or not (36 ± 3 μM) with EVs.

The above results show that i.v. injection of EVs from MSCs reduces neuroinflammation in hippocampus and restores cognitive function in hyperammonemic rats. To advance in the understanding of the mechanisms involved in the beneficial effects of EVs from MSCs, we used an ex vivo system allowing to analyze in detail the mechanisms involved. Freshly isolated hippocampal slices from hyperammonemic rats were treated ex vivo with EVs from MSCs. We first assessed if this ex vivo system reproduces the effects on neuroinflammation found in vivo.

### Ex vivo administration of EVs from MSCs reverses microglial and astrocytic activation in hippocampus of hyperammonemic rats and normalizes TNFα and IL-1β content

**Fig. 6:**
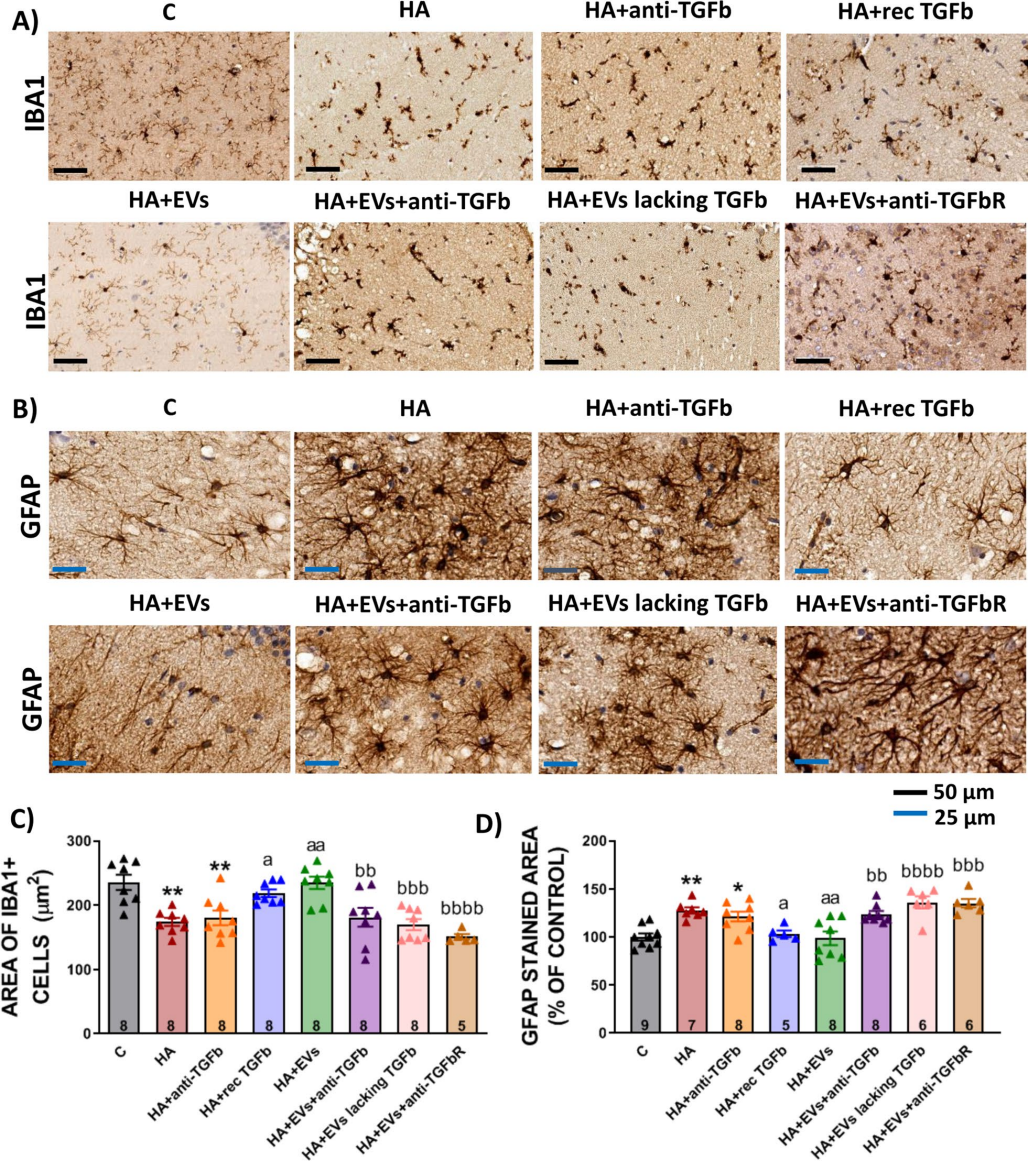
Incubation with EVs from MSCs reverses microglial and astrocytic activation in hippocampal slices from hyperammonemic rats. Representative images of **A** immunohistochemistry against Iba-1 and **B** GFAP in hippocampus. **C** Area of Iba1 stained cells (*n* = 5–8) and **D** percentage of area stained with GFAP, expressed as percentage of controls, (*n* = 5–9) in hippocampus. One-way ANOVA with Tukey post hoc test was performed to compare all groups. Values are the mean ± SEM. Values significantly different from controls are indicated by asterisk (*p < 0.05, **p < 0.01), values significantly different from HA group are indicated by a (a = *p* < 0.05, aa = *p* < 0.01) and values significantly different from HA + EVs group are indicated by b (bb = *p* < 0.01; bbb = *p* < 0.001; bbbb = *p* < 0.0001). Sample size of each group is indicated at the bottom of the bars

The area of microglial cells was reduced in hippocampal slices of hyperammonemic rats (174 ± 6 μm^2^ in comparison to 236 ± 12 μm^2^ in control slices, *p* < 0.01) and treatment with MSC-EVs reversed this effect (235 ± 10 μm^2^, *p* < 0.01) (Fig. 6A and C). The area stained with GFAP increased in hippocampal slices from hyperammonemic rats (128 ± 3%, *p* < 0.01), reflecting an astrocytes activation (Fig. 6B and D). This was reversed ex vivo by MSC-EVs (98 ± 7%, *p* < 0.01).

**Fig. 7:**
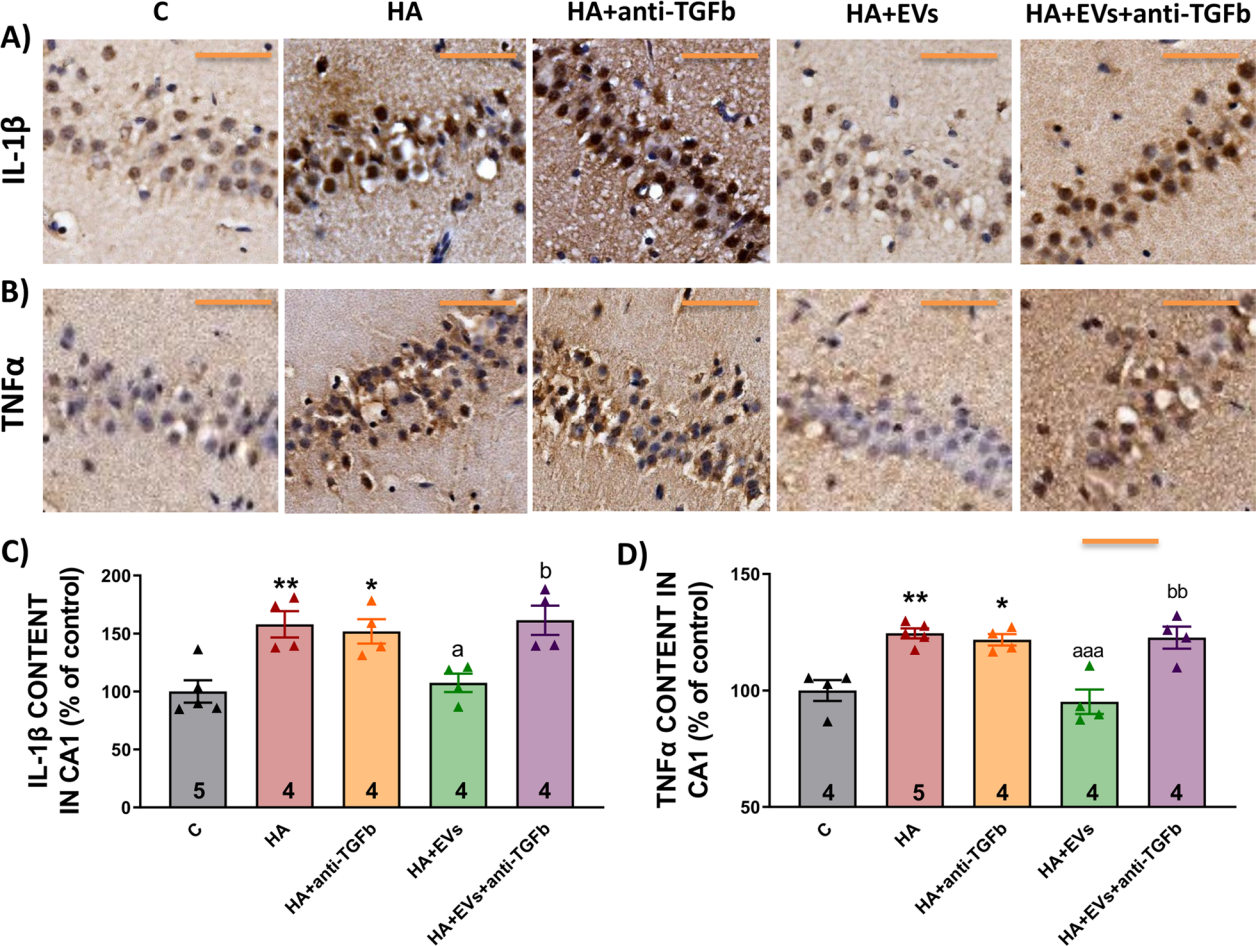
Incubation with MSC-EVs reduces the expression of pro-inflammatory markers IL-1β and TNFα in hippocampal slices from hyperammonemic rats. Representative images of **A** IL-1β and **B** TNFα in CA1 region. **C** Content of IL-1β (*n* = 4–5) and **D** TNFα (*n* = 4–5) in CA1 region of hippocampus, expressed as percentage of controls. One-way ANOVA with Tukey post hoc test was performed to compare all groups. Values are the mean ± SEM. Values significantly different from controls are indicated by asterisk (*p < 0.05; **p < 0.01), values significantly different from HA group are indicated by a (a = *p* < 0.05, aa = *p* < 0.01, aaa = *p* < 0.001) and values significantly different from HA + EVs group are indicated by b (b = *p* < 0.05; bb = *p* < 0.01). Sample size of each group is indicated at the bottom of the bars

The content of TNFα and IL-1β was increased in neurons of the CA1 region of hippocampus of hyperammonemic rats (158 ± 11%, *p* < 0.01; and 125 ± 2%, *p* < 0.01, respectively). Treatment with MSC-EVs normalized the amount of TNFα and IL-1β (107 ± 8%, *p* < 0.05; and 95 ± 5%, *p* < 0.05, respectively) (Fig. 7A–D). Therefore, the ex vivo system reproduces the effects of EVs from MSCs on neuroinflammation found in vivo.

### Ex vivo treatment with EVs induces a shift in hippocampus of hyperammonemic rats from a pro-inflammatory to an anti-inflammatory state

**Fig. 8:**
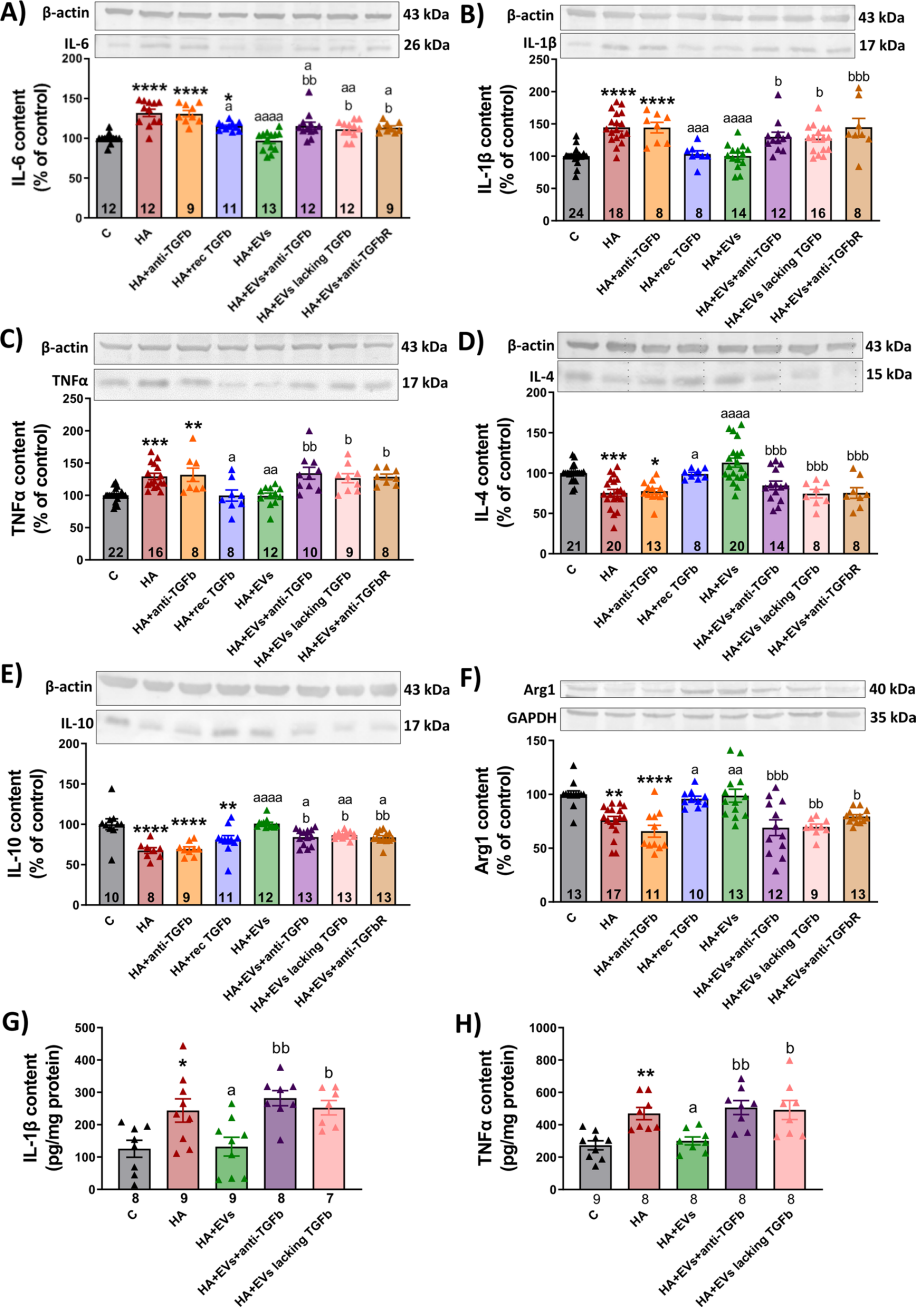
Incubation with MSC-EVs reduces the content of pro-inflammatory markers and restores the content of anti-inflammatory markers in hippocampal slices from hyperammonemic rats as measured by western blot. Content of **A** IL-6 (*n* = 9–13), **B** IL-1β (*n* = 8–24), **C** TNFα (*n* = 8–22), **D** IL-4 (*n* = 8–21), **E** IL-10 (*n* = 8–13) and **F** Arginase1 (*n* = 9–17) in homogenates from hippocampal slices measured by western blot. Representative images of the blots of each protein and the loading control (β-actin or GAPDH in case of Arginase1) are shown. Content of **G** IL-1β (*n* = 7–9) and **H** TNFα (*n* = 8–9) in homogenates from hippocampal slices measured by ELISA and expressed as pg per mg of total protein. One-way ANOVA with Tukey post hoc test was performed to compare all groups. Values are expressed as percentage of protein content in controls and are the mean ± SEM. Values significantly different from controls are indicated by asterisk (*p < 0.05; **p < 0.01; ***p < 0.001; ****p < 0.0001), values significantly different from HA group are indicated by a (a = *p* < 0.05; aa = *p* < 0.01; aaa = *p* < 0.001; aaaa = *p* < 0.0001) and values significantly different from HA + EVs group are indicated by b (b = *p* < 0.05; bb = *p* < 0.01; bbb = *p* < 0.001; bbbb = *p* < 0.0001). Sample size of each group is indicated at the bottom of the bars

The content of IL-6, IL-1β and TNFα were increased in hippocampal slices of hyperammonemic rats compared to control rats (132 ± 5%, *p* < 0.0001; 145 ± 6%, *p* < 0.0001; and 129 ± 5%, *p* < 0.001, respectively) as analyzed by Western blot (Fig. 8A–C). Treatment with MSC-EVs normalized the levels of these pro-inflammatory cytokines: IL-6 (97 ± 3%, *p* < 0.0001), IL-1β (100 ± 5%, *p* < 0.0001) and TNFα (100 ± 4%, *p* < 0.01). Similar results were obtained when the levels of IL-1β and TNFα were analyzed by ELISA. Hyperammonemia increases (*p* < 0.01) the content of IL-1β to 244 ± 36 pg/mg protein compared to 126 ± 26 pg/mg protein in control rats. Treatment with MSC-EVs normalized IL-1β levels to 132 ± 29 pg/mg protein (Fig. 8G). Hyperammonemia increases (*p* < 0.01) the content of TNFα to 470 ± 38 pg/mg protein compared to 273 ± 28 pg/mg protein in control rats. Treatment with MSC-EVs normalized TNFα levels to 300 ± 25 pg/mg protein (Fig. 8H).

The contents of IL-4 and IL-10 and arginase 1 were reduced in hippocampal slices from hyperammonemic rats (75 ± 4%, *p* < 0.001; 67 ± 4%, *p* < 0.0001; and 66 ± 6%, *p* < 0.0001) (Fig. 8D–F) and addition of MSC-EVs normalized them (100 ± 2%, *p* < 0.0001; 101 ± 2%, *p* < 0.0001; and 100 ± 6%, *p* < 0.01, respectively) These results show that the ex vivo system reproduces the effects on microglia polarization found in vivo in hyperammonemic rats injected with EVs from MSCs and it is therefore adequate to analyze the underlying mechanisms.

### TGFβ mediates the beneficial effects of MSC-EVs observed ex vivo

It has been proposed that MSCs modulates microglia activation via TGFβ secretion and also that EVs from MSCs contain TGFβ on their surface which mediates some beneficial effects of these EVs [62, 74, 86, 93, 94]. On the basis of these reports we hypothesized that the beneficial effects of EVs from MSCs on neuroinflammation in hyperammonemic rats would be mediated by TGFβ present in their membranes. To assess this possibility, we tested if the beneficial effects of EVs from MSCs in the ex vivo system are prevented by blocking TGFβ action by co-incubating with anti-TGFβ or by adding an antagonist of TGFβ receptor along with the EVs.

We also prepared MSCs lacking TGFβ (see “Materials and methods” section) and we assessed if the EVs from these MSCs lacking TGFβ loss their beneficial effects. Finally, we also assessed if the direct addition of recombinant TGFβ to the hippocampal slices from hyperammonemic rats reproduces the beneficial effects of EVs from MSCs.

As shown in Fig. 6, the capacity of EVs from MSCs to reverse microglia and astrocytes activation ex vivo was eliminated when the EVs were co-incubated with anti-TGFβ (area of microglia: 180 ± 17 μm^2^ versus 235 ± 10 μm^2^ in the HA + EVs group, *p* < 0.01; area stained with GFAP: 124 ± 8% versus 99 ± 7% in the HA + EVs group, *p* < 0.01) or when the EVs lacked TGFβ (area of microglia: 170 ± 9 μm^2^ versus 235 ± 10 μm^2^ in the HA + EVs group, *p* < 0.001; area stained with GFAP: 136 ± 6% versus 99 ± 7% in the HA + EVs group, *p* < 0.0001), thus supporting that TGFβ in the surface of the EVs is inducing these effects. This is further supported by the fact that addition of recombinant TGFβ to the hippocampal slices from hyperammonemic rats also reduced microglia (213 ± 10 μm^2^ versus 174 ± 6 μm^2^ in HA slices, *p* < 0.05) and astrocytes activation (103 ± 3% versus 128 ± 3% in HA slices, *p* < 0.05) similarly to MSC-EVs (Fig. 6). Slices from hyperammonemic rats incubated with anti-TGFβ showed microglia activation (180 ± 2 μm^2^ versus 236 ± 12 μm^2^ in control slices, *p* < 0.01) and astrocytes activation (121 ± 4% in comparison with control slices, *p* < 0.05) similar to slices of HA rats, indicating that the addition of anti-TGFβ to the slices did not have an effect on these parameters and that it was not responsible of the improvement observed in the slices treated with MSC-EVs previously incubated with anti-TGFβ (Fig. 6).

Similar results were obtained for inflammatory markers. Incubation of the MSC-EVs with anti-TGFβ prevented the reduction by EVs of IL-1β (161 ± 13% versus 107 ± 8% in the HA + EVs group, *p* < 0.05) and TNFα (123 ± 5% versus 95 ± 5% in the HA + EVs group, *p* < 0.01) in hippocampal neurons of CA1 region, as assessed by immunohistochemistry (Fig. 7). Incubation of slices from hyperammonemic rats with anti-TGFβ did not affect the levels of IL-1β (153 ± 11% of control, *p* < 0.05) or TNFα (122 ± 2% of control, *p* < 0.05), which were increased in comparison to slices from control rats.

TGFβ is also responsible for the shift from pro-inflammatory to anti-inflammatory induced by EVs from MSCs in hippocampus of hyperammonemic rats (Fig. 8). Co-incubation with anti-TGFβ prevented the reduction by EVs of the levels of pro-inflammatory cytokines IL-6 (116 ± 5%, *p* < 0.01) (Fig. 8A), IL-1β (130 ± 7%, *p* < 0.05) (Fig. 8B) and TNFα (134 ± 9%, *p* < 0.01) (Fig. 8C) as well as the increase of the anti-inflammatory IL-4 (85 ± 5%, *p* < 0.001) (Fig. 8D), IL-10 (84 ± 3%, *p* < 0.05) (Fig. 8E) and arginase 1 (70 ± 7%, *p* < 0.001) (Fig. 8F). Depletion of TGFβ from the MSCs also prevented the effects of MSC-EVs on these pro-inflammatory (IL-6: 112 ± 3%, *p* < 0.05; IL-1β: 127 ± 5%, *p* < 0.05; TNFα: 127 ± 7%, *p* < 0.05) and anti-inflammatory factors (IL-4: 74 ± 6%, *p* < 0.001; IL-10: 87 ± 1%, *p* < 0.05; Arginase 1: 70 ± 3%, *p* < 0.01) as assessed by Western blot (Fig. 8A-F). Similar results were obtained when IL-1β and TNFα were analyzed by ELISA. MSCs-EVs reduced the levels of IL-1β in hyperammonemic rats from 244 ± 36 to 132 ± 29 pg/mg protein; however, treatment with TGFβ-depleted EVs did not reduce IL-1β, maintaining it at 253 ± 22 pg/mg protein (Fig. 8G). EVs reduced the levels of TNFα in hyperammonemic rats from 470 ± 38 to 300 ± 25 pg/mg protein; however, treatment with TGFβ-depleted EVs maintained TNFα at 492 ± 58 pg/mg protein (Fig. 8H).

Moreover, addition of recombinant TGFβ was also able to induce the shift to the anti-inflammatory state, reducing IL-6 (115 ± 2%, *p* < 0.05), IL-1β (103 ± 5%, *p* < 0.001) and TNFα (100 ± 10%, *p* < 0.05) and increasing IL-4 (99 ± 2%, *p* < 0.05), IL-10 (81 ± 5%, *p* < 0.01) and arginase 1 (96 ± 2%, *p* < 0.05) (Fig. 8A–F). These data indicate that TGFβ in the surface of the EVs is responsible for the reduction of glial activation and neuroinflammation induced by EVs from MSCs.

### Ex vivo administration of MSC-EVs reverses the alterations in membrane expression of AMPA and NMDA receptors in hippocampal slices from hyperammonemic rats

Hernandez-Rabaza et al. [40], Taoro-Gonzalez et al. [78, 79] and Balzano et al. [9] have shown that neuroinflammation induces alterations in the membrane expression of AMPA (GluA1 and GluA2) and NMDA (NR2B) receptor subunits in hippocampus, which are responsible for the impairment of spatial learning in hyperammonemic rats and that treatments that normalize membrane expression of these subunits restore cognitive function.

**Fig. 9:**
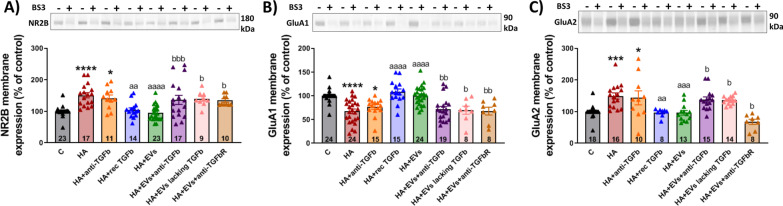
Incubation with MSC-EVs normalizes the membrane expression of NR2B subunit of NMDA receptors and GluA1 and GluA2 subunits of AMPA receptors in hippocampal slices from hyperammonemic rats. Membrane expression of **A** NR2B (*n* = 9–23), **B** GluA1 (*n* = 8–24) and **C** GluA2 (*n* = 8–18) in homogenates from hippocampal slices incubated in the presence (+) or absence (−) of the cross-linker BS3, measured by western blot. Samples in the absence of BS3 represent the total amount of each protein, while samples incubated in the presence of BS3 represent the non-membrane fraction of each protein. Representative images of the blots of each protein are shown. One-way ANOVA with Tukey post hoc test was performed to compare all groups. Values are expressed as percentage of membrane expression of controls and are the mean ± SEM. Values significantly different from controls are indicated by asterisk (*p < 0.05; ***p < 0.001; ****p < 0.0001), values significantly different from HA group are indicated by a (aa = *p* < 0.01; aaa = *p* < 0.001; aaaa = *p* < 0.0001) and values significantly different from HA + EVs group are indicated by b (b = *p* < 0.05; bb = *p* < 0.01; bbb = *p* < 0.001). Sample size of each group is indicated at the bottom of the bars

We therefore assessed using the cross-linker BS3 if addition of EVs from MSCs to hippocampal slices from hyperammonemic rats normalizes membrane expression of AMPA and NMDA receptors subunits. Hyperammonemia increased membrane expression of the NR2B subunit of NMDA receptors (151 ± 8%, *p* < 0.0001) (Fig. 9A) and of the GluA2 subunit of AMPA receptors (150 ± 10%, *p* < 0.001) (Fig. 9C) and reduced membrane expression of the GluA1 subunit of AMPA receptors (67 ± 5%, *p* < 0.0001) (Fig. 9B) in the hippocampal slices. Treatment with EVs from MSCs normalized the membrane expression of NR2B (96 ± 5%, *p* < 0.0001), GluA1 (100 ± 4%, *p* < 0.0001) and GluA2 (96 ± 7%, *p* < 0.001) subunits (Fig. 9). This normalization of membrane expression of AMPA and NMDA receptor subunits would mediate the restoration of cognitive function. The normalization of membrane expression of NR2B, GluA1, and GluA2 did not occur in the presence of anti-TGFβ (136 ± 15%, *p* < 0.01; 71 ± 6, *p* < 0.01; and 138 ± 8%, *p* < 0.05, respectively) or when EVs lacking TGFβ were used (140 ± 8%, *p* < 0.05; 70 ± 9%, *p* < 0.05; and 136 ± 4%, *p* < 0.05, respectively). Conversely, the normalization induced by MSC-EVs was mimicked by addition of recombinant TGFβ (NR2B: 104 ± 7%, *p* < 0.01); GluA1: 108 ± 6%, *p* < 0.0001; and GluA2: 97 ± 5%, *p* < 0.01) (Fig. 9).

This indicates that TGFβ in the surface of MSCs-EVs is responsible for the normalization of membrane expression of NMDA and AMPA receptor subunits, which in turn would be responsible for restoration of learning and memory in hyperammonemic rats.

### Incubation with MSC-EVs reduces NF-κB activation in hippocampal slices from hyperammonemic rats through the TGFβ–TGFβR2–Smad7–IkBα pathway

To further advance in the understanding of the mechanisms by which EVs from MSCs reduce neuroinflammation in hippocampus of hyperammonemic rats, we assessed whether nuclear translocation of NF-κB is increased in hippocampal slices from hyperammonemic rats and if this is reversed by MSC-EVs. Dadsetan et al. [22] reported that in rats with porta-cava shunts, another model of MHE, the increased levels of IL-1β and TNFα in hippocampus are a consequence of increased activation and nuclear translocation of NF-κB.

**Fig. 10:**
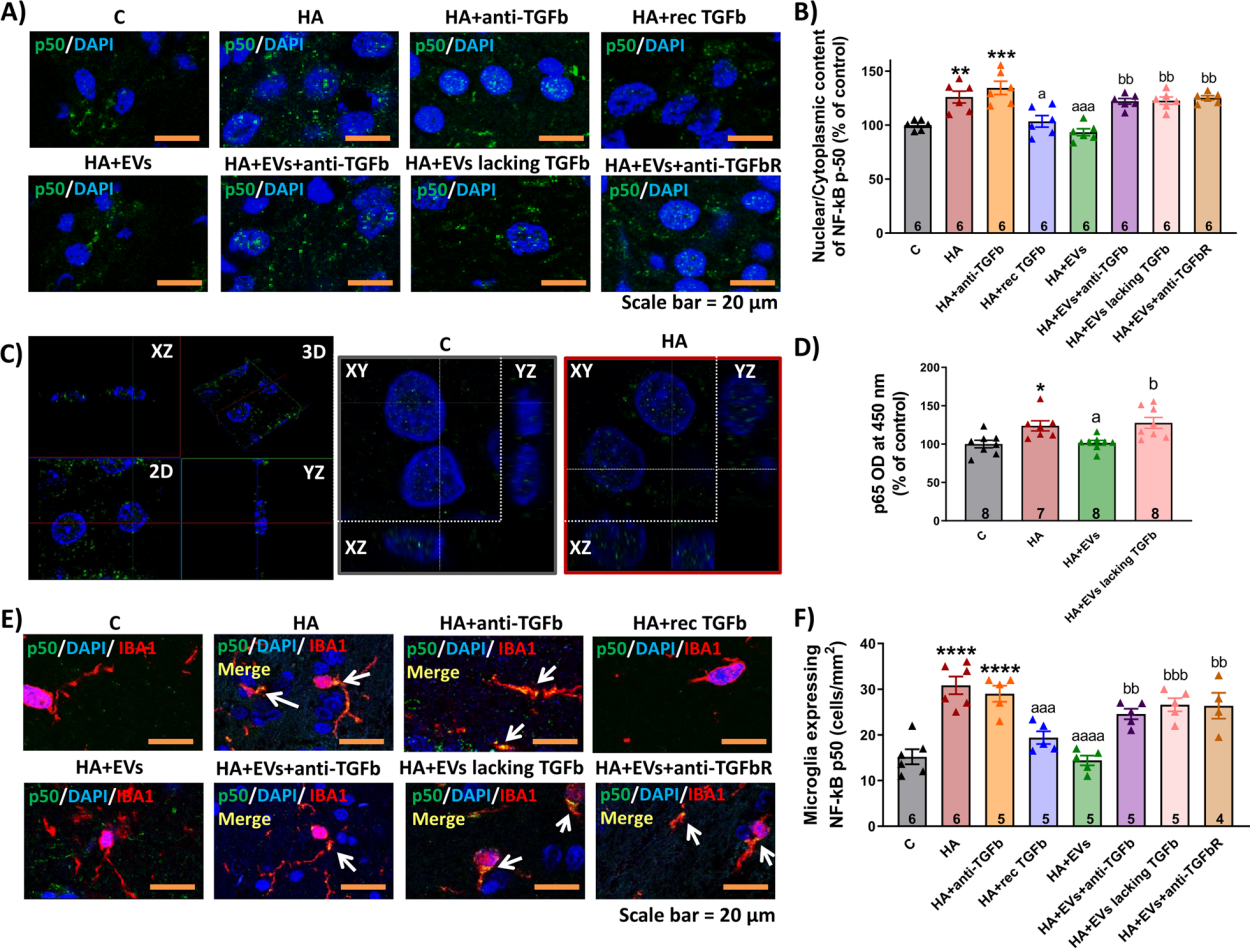
Incubation with MSC-EVs reduces NF-κB activation in hippocampal slices from hyperammonemic rats. **A** Representative images of immunofluorescence against p50 subunit of NF-κB (green) in hippocampal slices. Nuclei are stained with DAPI (blue). **B** Ratio of nuclear/cytoplasmic NF-κB p50 subunit in neurons of CA1 region of hippocampus, measured by immunofluorescence and expressed as percentage of control (*n* = 5–6). **C** Axial projections of z-stack to confirm *p*-50 nuclear localization: a representative image showing 3D and 2D projections with its corresponding XZ and YZ planes is shown on the left. Representative images of control and HA samples showing 2D projections with their corresponding XZ and YZ planes are shown on the right. **D** p65 transcriptional activity in nuclear extracts measured by DNA-binding activity kit. Data of optical density were measured at 450 nm and are expressed as percentage of controls. **E** Double-immunofluorescence against p50 subunit of NF-kB (green) and Iba1 (staining microglia, in red). Nuclei are stained with DAPI (blue). **F** Number of microglial cells expressing NF-κB p50 subunit, measured by double immunofluorescence and expressed as cells/mm^2^ (*n* = 4–6). One-way ANOVA with Tukey post hoc test was performed to compare all groups. Values are the mean ± SEM. Values significantly different from controls are indicated by asterisk (*p < 0.05; **p < 0.01; ***p < 0.001; ****p < 0.0001), values significantly different from HA group are indicated by a (a = *p* < 0.05; aaa = *p* < 0.001; aaaa = *p* < 0.0001) and values significantly different from HA + EVs group are indicated by b (b = *p* < 0.05; bb = *p* < 0.01; bbb = *p* < 0.001). Sample size of each group is indicated at the bottom of the bars

The nuclear content of the p50 subunit of NF-κB was increased in hippocampal neurons of CA1 region in slices from hyperammonemic rats (126 ± 6% of control, *p* < 0.01) (Fig. 10A and B) and the number of microglial cells expressing NF-κB was also increased (31 ± 2 cells/mm^2^ versus 15 ± 2 cells/mm^2^ in control slices, *p* < 0.0001) (Fig. 10E and F) and these increases were reversed by EVs from MSCs (nuclear/cytoplasmic content of p50: 93 ± 4%, *p* < 0.001; and microglia expressing p50: 14 ± 1 cells/mm^2^, *p* < 0.0001). The normalization of nuclear NF-κB did not occur if EVs from MSCs were added in the presence of anti-TGFβ (nuclear/cytoplasmic content of p50: 122 ± 4%, *p* < 0.01; and microglia expressing p50: 25 ± 1 cells/mm^2^, *p* < 0.01) or if the EVs were depleted of TGFβ (nuclear/cytoplasmic content of p50: 123 ± 3%, *p* < 0.01; and microglia expressing p50: 27 ± 1 cells/mm^2^, *p* < 0.001) (Fig. 10A, B). Incubation of the hippocampal slices from hyperammonemic rats with anti-TGFβ did not prevent the increase in p50 nuclear content (133 ± 8%, *p* < 0.001 compared to control slices) or in the microglia expressing p50 (29 ± 2 cells/mm^2^, *p* < 0.0001 compared to control slices). Moreover, addition of recombinant TGFβ reproduced the effects of EVs (nuclear/cytoplasmic content of p50: 104 ± 5%, *p* < 0.05; and microglia expressing p50: 19 ± 1 cells/mm^2^, *p* < 0.001), indicating that TGFβ in the EVs is responsible for this effect. Figure 10C shows axial projections of z-stack taken to confirm that p50 staining was localized in the nuclei.

To corroborate the effects on NF-κB activation, p65 NF-κB transcriptional activity was measured in nuclear extracts using a commercial kit. The results show that hyperammonemia increases p65 activity in nuclear extracts from hippocampal slices (124 ± 7% of control, *p* < 0.05) and treatment with MSC-EVs reverses this activation (102 ± 3%, *p* < 0.05). In contrast, MSC-EVs depleted of TGFβ did not reduce p65 activity (128 ± 7%, *p* < 0.05) (Fig. 10D).

We then tried to understand how TGFβ reduces NF-κB signaling. Noh et al. [62] reported that MSC-secreted TGFβ inhibits the NF-κB pathway in LPS-activated microglia by modulating Smad2/3 phosphorylation through the TGFβ1 receptor. We therefore tested if the Smad2/3 pathway could be mediating the effects of EVs TGFβ on NF-κB signaling in hippocampal slices of hyperammonemic rats. We did not find any change in the phosphorylation of Smad2 or Smad3 in hippocampal slices from hyperammonemic rats. Moreover, treatment with EVs from MSCs did not affect either Smad2 or Smad 3 phosphorylation (not shown). This indicates that the TGFβ–Smad2/3 pathway is not involved in the beneficial effects of EVs from MSCs.

**Fig. 11:**
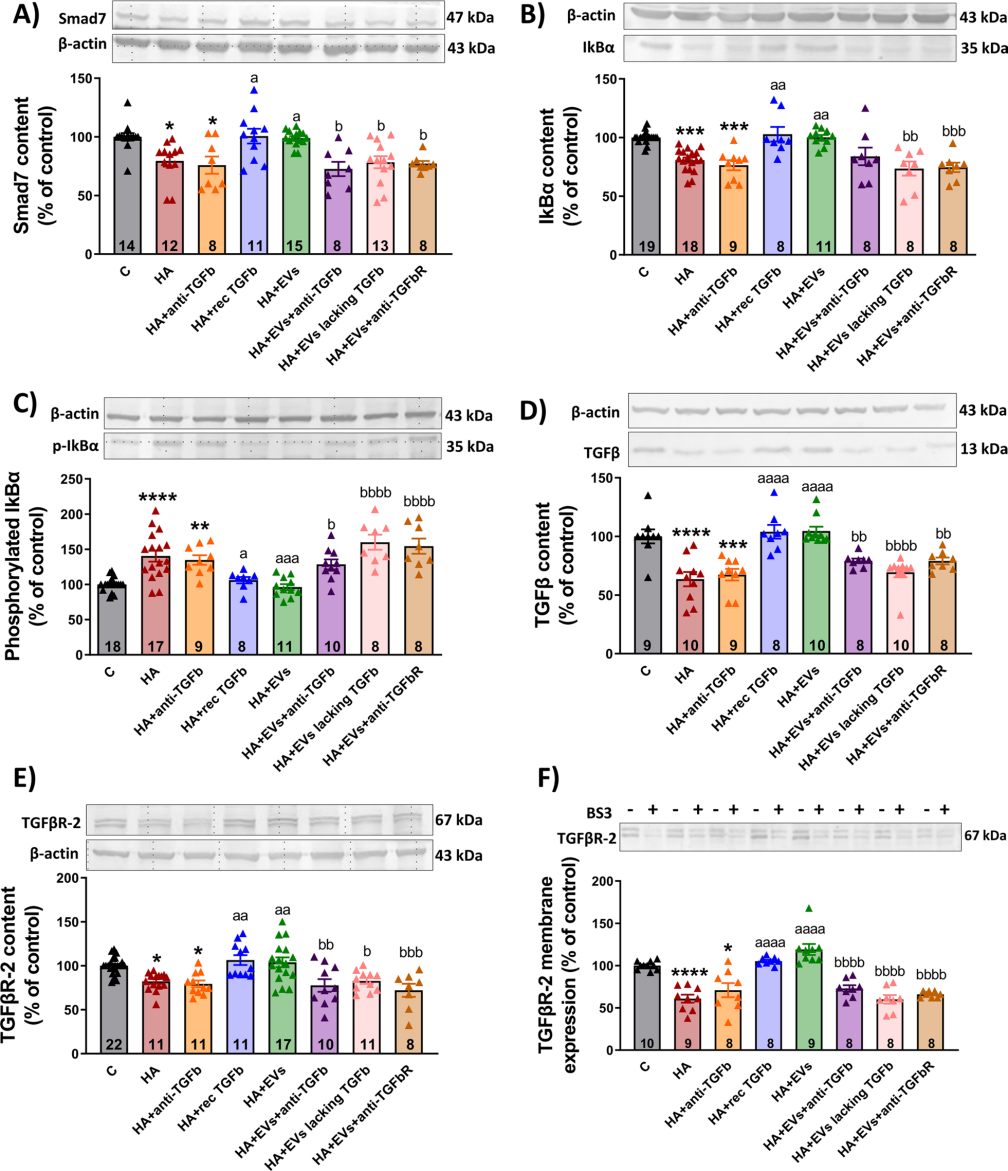
Incubation with MSC-EVs reduces NF-κB activation in hippocampal slices from hyperammonemic rats through the TGFβ–TGFβR2–Smad7–IkBα pathway. Content of **A** Smad7 (*n* = 8–15), **B** IkBα (*n* = 8–19), **C** phosphorylated IkBα (*n* = 8–18), **D** TGFβ (*n* = 8–10) and **E** TGFβR2 (*n* = 8–22) in homogenates from hippocampal slices, measured by western blot and expressed as percentage of protein content in controls. Representative images of the blots of each protein and the load control (β-actin) are shown. **F** Membrane expression of TGFβR2 (*n* = 8–10) in homogenates from hippocampal slices incubated in the presence (+) or absence (−) of the cross-linker BS3, measured by western blot. Samples in the absence of BS3 represent the total amount of each protein, while samples incubated in the presence of BS3 represent the non-membrane fraction of each protein. Representative images of the blots are shown. One-way ANOVA with Tukey post hoc test was performed to compare all groups. Values are the mean ± SEM. Values significantly different from controls are indicated by asterisk (*p < 0.05; **p < 0.01; ***p < 0.001; ****p < 0.0001), values significantly different from HA group are indicated by a (a = *p* < 0.05; aa = *p* < 0.01; aaa = *p* < 0.001; aaaa = *p* < 0.0001) and values significantly different from HA + EVs group are indicated by b (b = *p* < 0.05; bb = *p* < 0.01; bbb = *p* < 0.001; bbbb = *p* < 0.0001). Sample size of each group is indicated at the bottom of the bars

It has been shown that TGFβ may also inhibit NF-κB signaling by inducing Smad7, which enhances the transcription of IkB, a key inhibitor of NF-κB signaling pathway. Smad7 may also disrupt the TRAF–TAK1–TAB2/3 complex, thus inhibiting NF-κB signaling [91]. We therefore assessed if the Smad7–IkB pathway could be mediating the effects of EVs TGFβ on NF-κB signaling in hippocampal slices of hyperammonemic rats. We found that hyperammonemia reduced Smad7 content in hippocampus (79 ± 5% of control, *p* < 0.05) (Fig. 11A) and this is associated with a parallel reduction of the IkB content (81 ± 2% of control, *p* < 0.001) (Fig. 11B). Moreover, hyperammonemia also increased the phosphorylation of IkB (140 ± 8% of control, *p* < 0.0001) (Fig. 11C). All these factors would contribute to enhanced nuclear translocation of NF-κB and activation of NF-κB signaling, including transcription of IL-1β and TNFα.

Treatment of the hippocampal slices from hyperammonemic rats with EVs from MSCs normalized the levels of Smad7 (99 ± 2%, *p* < 0.05) and IkB (100 ± 7%, *p* < 0.01), as well as the phosphorylation of IkB (100 ± 4%, *p* < 0.001), which returned to values similar to control rats (Fig. 11A–C). Normalization of these parameters did not occur if EVs were added in the presence of anti-TGFβ (Smad7: 73 ± 7%, *p* < 0.01; IkB: 84 ± 9%, *p* = 0.1; and phospho-IkB: 130 ± 7%, *p* < 0.05) or if TGFβ-depleted EVs were used (Smad7: 78 ± 5%, *p* < 0.05; IkB: 73 ± 8%, *p* < 0.01; and phospho-IkB: 160 ± 20%, *p* < 0.0001), while the addition of anti-TGFβ alone did not alter them (Smad7: 73 ± 7%, *p* < 0.01; IkB: 84 ± 9%, *p* = 0.1; and phospho-IkB: 130 ± 7%, *p* < 0.05). Moreover, the levels of Smad7 and IkB, and phosphorylation of IkB were also normalized if recombinant TGFβ was added to the hippocampal slices from hyperammonemic rats (Smad7: 76 ± 7%, *p* < 0.05; IkB: 76 ± 4%, *p* < 0.001; and phospho-IkB: 130 ± 7%, *p* < 0.0001) (Fig. 11A–C).

These data support the idea that TGFβ in the surface of EVs from MSCs reverses the enhanced NF-κB signaling in hippocampus of hyperammonemic rats by normalizing the levels of Smad7 and IkB.

We then assessed if the reduced levels of Smad7 and IkB in hippocampus of hyperammonemic rats could be due to reduced levels of TGFβ, or to reduced content or membrane expression of its receptors. The content of TGFβ was reduced (64 ± 6%, *p* < 0.0001) in hippocampal slices from hyperammonemic rats and was restored to normal levels by treatment with EVs from MSCs (105 ± 4%, *p* < 0.0001) or with recombinant TGFβ (104 ± 7%, *p* < 0.0001), but not by EVs co-incubated with anti-TGFβ (79 ± 2%, *p* < 0.01) or EVs lacking TGFβ (69 ± 4%, *p* < 0.0001) (Fig. 11D). Hyperammonemia also reduced the total content (82 ± 3%, *p* < 0.05) (Fig. 11E) and membrane expression (54 ± 6%, *p* < 0.0001) (Fig. 11F) of the TGFβ receptor 2, which were also normalized by treatment with EVs from MSCs (104 ± 6%, *p* < 0.01; and 119 ± 10%, *p* < 0.0001, respectively) or with recombinant TGFβ (107 ± 6%, *p* < 0.01; and 105 ± 2%, *p* < 0.0001, respectively) but not by EVs in the presence of anti-TGFβ (78 ± 7%, *p* < 0.01; and 73 ± 6%, *p* < 0.0001, respectively) or by EVs lacking TGFβ (83 ± 3%, *p* < 0.05; and 60 ± 7%, *p* < 0.0001, respectively) (Fig. 11E, F).

To confirm that the beneficial effects of EVs from MSCs are mediated by activation of TGFβ receptors by TGFβ present in the membrane surface of EVs, we assessed if these beneficial effects were prevented by blocking TGFβ receptors 1 and 2 with a selective antagonist. The results show that this is the case. Blocking TGFβ receptors also prevents the beneficial effects of EVs from MSCs on neuroinflammation, preventing the reduction of NF-κB activation in neurons (p50 nuclear content: 125 ± 2%, *p* < 0.01) (Fig. 10A, B) and microglia (microglia expressing p50: 26 ± 3 cells/mm^2^, *p* < 0.01) (Fig. 10E, F), the normalization of Smad7 (77 ± 3%, *p* < 0.05), IkB (75 ± 4%, *p* < 0.001) and *p*-IkB (150 ± 10%, *p* < 0.0001) (Fig. 11A–C), of TGFβ levels (79 ± 3%, *p* < 0.01) (Fig. 11D) and of TGFβ receptor 2 amount (83 ± 3%, *p* < 0.001) and membrane expression (66 ± 2%, *p* < 0.0001) (Fig. 11E, F). Blocking TGFβ receptors also prevents the reduction of microglial (area of the microglial cells 151 ± 4 μm^2^ versus 235 ± 10 μm^2^ in the slices treated with EVs, *p* < 0.05) and astrocytes activation (135 ± 4%, *p* < 0.001) (Fig. 6) and the shift from pro- to anti-inflammatory state induced by EVs, preventing the changes in IL-6 (114 ± 2%, *p* < 0.05), IL-1β (145 ± 14%, *p* < 0.001), TNFα (129 ± 4%, *p* < 0.05), IL-4 (75 ± 7%, *p* < 0.001), IL-10 (84 ± 2%, *p* < 0.01) and arginase 1 (79 ± 2%, *p* < 0.05) (Fig. 8).

## Discussion

This study shows that EVs from MSCs injected to hyperammonemic rats reach the hippocampus and reduce glial activation and neuroinflammation and restore cognitive function in hyperammonemic rats. Moreover, as discussed below, the study also unveils the underlying mechanisms involved in these beneficial effects of EVs from MSCs and supports the idea that these EVs may be a good therapeutic agent to reverse cognitive impairment in cirrhotic patients with minimal or clinical hepatic encephalopathy.

Hyperammonemia is a main contributor to the neurological (both cognitive and motor) alterations in patients with MHE or clinical HE [27, 30, 72]. In fact, in the last decades the main treatments of these patients aim to reduce ammonia levels using lactulose, reducing protein intake or by other means [32, 99]. In the last decade, it has been shown that the deleterious effects of hyperammonemia on cognitive and motor function are mediated by induction of neuroinflammation, which alters neurotransmission, leading to the impairment of cognitive and motor function (reviewed by Cabrera-Pastor et al. [14]). Studies in animal models of hyperammonemia and MHE show that cognitive and motor function may be restored by different pharmacological approaches acting on different steps of pathways involved in inflammation, neuroinflammation or GABAergic neurotransmission [1, 12, 13, 17, 21, 22, 39–41, 44, 57, 66]. However, most of these treatments would have secondary effects if used in patients with liver cirrhosis and MHE or clinical HE. For example, non-steroidal anti-inflammatory drugs such as ibuprofen may induce serious renal problems in cirrhotic patients [19] or inhibitors of phosphodiesterase 5 could aggravate hemodynamic problems in advanced cirrhosis [87]. Therefore, safe procedures to reduce neuroinflammation without inducing secondary effects are needed to treat patients with hyperammonemia and MHE or clinical HE.

A promising approach to reduce neuroinflammation in different types of pathologies is the use of EVs from MSCs. EVs are natural carrier systems that transfer information from the original cells to the recipient cells mainly through transmission of microRNAs or proteins. A main advantage of EVs as therapeutic agents is that they can cross the blood–brain barrier and transfer the information to brain cells, thus avoiding the problems of restriction of transport through the blood–brain barrier that present many pharmacological drugs. Intravenously injected EVs reach different organs, including brain, liver, spleen, heart, lungs and gastrointestinal tract [52, 63, 75, 89]. EVs from MSCs also induce beneficial effects in other pathologies such as liver or kidney diseases [48, 51].

Mesenchymal stem cells modulate the innate and adaptive immune system and show potent anti-inflammatory properties which have motivated their use in many clinical trials to treat different pathologies [58]. Recent studies indicate that EVs from MSCs may induce similar beneficial effects with lower immunogenic response and easier transport to brain [5, 54, 59, 63, 70].

EVs from MSCs have been shown to attenuate neuroinflammation evoked by ischemic brain injury [20, 34, 45], perinatal brain injury [81] and may be also useful in models of Alzheimer’s disease [64], Parkinson’s disease [85] or multiple sclerosis [71].

We show here that EVs from MSCs also reduce neuroinflammation and restore cognitive function in rats with chronic hyperammonemia, a main contributor to MHE and clinical HE. The results reported support the idea that EVs from MSCs would also improve cognitive function in patients with liver cirrhosis and MHE or clinical HE.

**Fig. 12:**
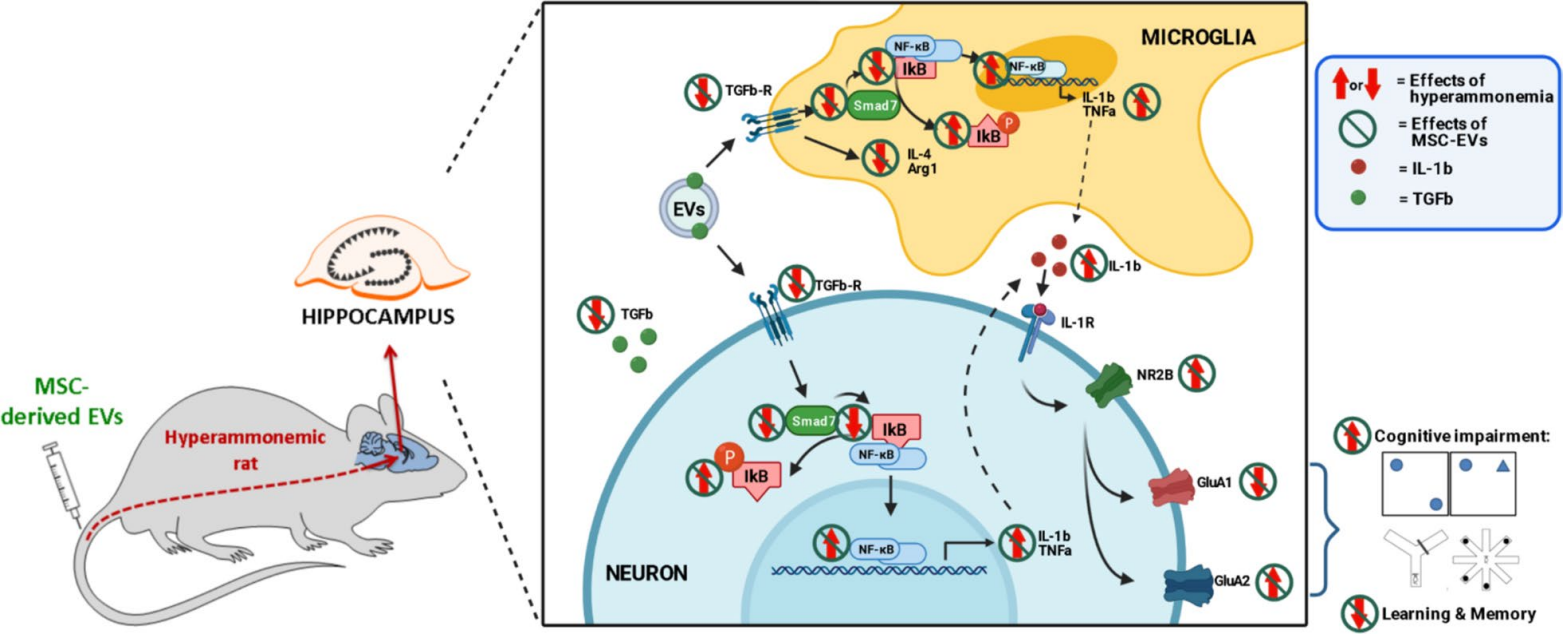
Summary of the main effects of MSC-EVs in hippocampus of hyperammonemic rats: underlying mechanisms. Hyperammonemia induces neuroinflammation in hippocampus, with microglial activation, increasing pro-inflammatory factors (IL-1β, TNFα) and reducing anti-inflammatory factors (IL-4, Arg1). Increased IL-1β and activation of its receptor alters membrane expression of NR2B, GluA1 and GluA2 subunits of NMDA and AMPA receptors, leading to impairment of cognitive function, and to learning and memory deficits. Extracellular vesicles derived from mesenchymal stem cells (MSCs) injected to hyperammonemic rats reach the hippocampus, reduce the expression of pro-inflammatory factors and increase the expression of anti-inflammatory factors, reverse neuroinflammation in hippocampus and restore different forms of learning and memory. The results reported indicate that these beneficial effects are mediated by the TGFβ–TGFβR2–Smad7–IkBα–NF-κB pathway. The content of TGFβ, its receptor TGFβR2 and Smad7 are reduced in hyperammonemia, leading to reduced IkBα protein and increased NF-κB activation, which induces the expression of pro-inflammatory markers such as IL-1β and TNFα, leading to cognitive impairment. EVs from MSCs contain TGFβ, which normalizes the TGFβ–TGFβR2–Smad7–IkBα–NF-κB pathway in hyperammonemic rats. This normalizes IL-1β levels and, subsequently the membrane expression of NR2B, GluA1 and GluA2 subunits, restoring cognitive function. *Created with Biorender*

Moreover, the study also unveils the mechanisms involved in these beneficial effects of EVs from MSCs. These mechanisms are summarized in Fig. 12. Impairment of cognitive functions modulated by hippocampus in hyperammonemic rats are a consequence of altered membrane expression of the GluA1 and GluA2 subunits of AMPA receptors, which in turn are due to increased membrane expression of the NR2B subunit of NMDA receptors [9, 14, 39, 40, 78, 79].

In this study hyperammonemic rats show impaired reference memory (a spatial memory), and a tendency to commit more working errors (a non-spatial memory) that did not reach statistical significance. The mechanisms and circuits involved in modulation of spatial and non-spatial memory are different. Spatial memory is modulated by postrhinal-to-hippocampus circuits, while non-spatial memory is modulated by perirhinal-to-hippocampus circuits [84]. Taoro-Gonzalez et al. [80] observed that these circuits would be differently affected by hyperammonemia and Cabrera-Pastor et al. [11] suggested a dissociation between different inflammatory factors and their contribution to different types of cognitive alterations, since the administration of extracellular cGMP to hyperammonemic rats reversed reference memory impairment but not working memory impairment. These differences in the mechanisms modulating both aspects of memory in HA rats may explain the different effects observed for reference and working memory.

We show here that EVs from MSCs restore learning and memory of hyperammonemic rats in different tasks modulated by hippocampus (object location and object recognition memory test, short-term memory in the Y-maze and learning and reference memory in the radial maze). Restoration of learning and memory was associated with a normalization of membrane expression of the GluA1 and GluA2 subunits of AMPA receptors, which would be the cause for the recovery of learning and memory.

The altered membrane expression of GluA1, GluA2 and NR2B in hippocampus of hyperammonemic rats is a consequence of increased activation of the IL-1 receptor by the increased levels of IL-1β [78]. EVs from MSCs normalize membrane expression of AMPA and NMDA receptors by normalizing IL-1β levels.

The increase in IL-1β in hippocampus of hyperammonemic rats has a dual origin. Part of the IL-1β is synthesized in activated microglia and part in neurons (Fig. 12). EVs from MSCs normalize IL-1β production both in microglia and in neurons.

In microglia, a main mechanism by which EVs reduce IL-1β levels is by reducing microglia activation. EVs from MSCs reduce microglial activation and induce a switch in the phenotype of microglia in hippocampus of hyperammonemic rats, from a classically activated phenotype with increased levels of IL-1β and IL-6 and reduced levels of IL-4 and arginase 1 to an anti-inflammatory phenotype with normalized levels of these cytokines. This is associated with a reduction of NF-κB levels, which would reduce transcription of pro-inflammatory proteins. This effect is similar to the phenotype switch described by Noh et al. [62], who reported that MSCs modulate the functional properties of microglia via TGFβ secretion, switching them from a classically activated phenotype to an inflammation-resolving phenotype. This suggests that TGFβ in the surface of EVs would mediate the switch in the phenotype of microglia in hippocampus of hyperammonemic rats. This is further supported by the fact that deactivation of microglia by EVs from MSCs is prevented by anti-TGFβ or in EVs lacking TGFβ and is reproduced by direct addition of TGFβ to the hippocampal slices. Activation of TGFβ receptors in microglia by TGFβ in the surface of EVs from MSCs would therefore trigger the shift to reduce the pro-inflammatory state in hippocampus of hyperammonemic rats. This is further supported by the fact that blocking TGFβ receptors with a selective antagonist prevents the induction of the shift. This shift would be associated with a reduced synthesis of IL-1β in microglia.

Concerning neurons, the increased transcription of IL-1β in hyperammonemia and MHE is due to increased nuclear translocation of NF-κB, which promotes transcription of IL-1β, TNFα and other pro-inflammatory factors [22]. EVs from MSCs reduces activation of NF-κB signaling in neurons in hippocampal slices from hyperammonemic rats by reducing the nuclear translocation of NF-κB, thus reducing to normal levels the amounts of IL-1β, TNFα and other pro-inflammatory factors which transcription is promoted by NF-κB.

The normalization of IL-1β levels in microglia and neurons reverses the enhanced activation of IL-1 receptor in hippocampus of hyperammonemic rats, thus normalizing membrane expression of GluA1, GluA2 and NR2B and cognitive function.

We assessed how EVs reduces NF-κB translocation to the nucleus and, therefore, all subsequent events mentioned above and summarized in Fig. 12.

Noh et al. [62] proposed that MSCs can modulate the functional properties of microglia via TGFβ secretion, switching them from a classically activated phenotype to an inflammation-resolving phenotype. EVs from MSCs and from other cell types contain TGFβ on their surfaces [74, 86, 94]. This TGFβ seems to mediate some of the beneficial effects of EVs [76, 97, 98]. Exosomes expressing TGFβ in their membranes show a potent immunosuppressive activity and inhibit murine experimental autoimmune encephalomyelitis (EAE), a model for multiple sclerosis [94]. Exosomes derived from MSCs reverse epithelial–mesenchymal transition via TGFβ/Smad pathway and promote repair of damaged endometrium [93].

On the basis of these reports, we hypothesized that the reversal by EVs from MSCs of increased nuclear translocation of NF-κB in hippocampal neurons of hyperammonemic rats would be mediated by TGFβ present in the EVs membranes. We performed experiments showing that the reversal of nuclear translocation of NF-κB by EVs is prevented by anti-TGFβ or by blocking TGFβ receptors, is not induced by EVs from MSCs lacking TGFβ and is mimicked by addition of recombinant TGFβ. This supports that the reversal of increased nuclear translocation of NF-κB in neurons by EVs from MSCs is mediated by TGFβ in the surface of the EVs. This would be associated with a reduced synthesis of IL-1β and other pro-inflammatory markers in hippocampal neurons of hyperammonemic rats.

We then tried to understand how TGFβ reduces NF-κB signaling. Noh et al. [62] reported that MSC-secreted TGF-b inhibits the NF-κB pathway in LPS-activated microglia by modulating Smad2/3 phosphorylation through the TGFβ1 receptor. Activation of the TGFβ–Smad2 pathway is also involved in the differentiation of umbilical cord-derived MSCs to carcinoma-associated fibroblasts induced by gastric cancer exosomes [36]. We therefore tested if the Smad2/3 pathway could be mediating the effects of EVs TGFβ on NF-κB signaling in hippocampal slices of hyperammonemic rats, but, as indicated in the Results section, this was not the case.

TGFβ may also inhibit NF-κB signaling by inducing Smad7, which enhances the transcription of IkB, a key inhibitor of NF-κB signaling pathway. Smad7 may also disrupt the TRAF–TAK1–TAB2/3 complex, thus inhibiting NF-κB signaling [91]. We show that hyperammonemia reduces the content of Smad7 and IkB in hippocampus, which are restored by treatment with EVs from MSCs and also by treatment with recombinant TGFβ. This suggests that normalization of the Smad7–IkB pathway would be mediating the effects of TGFβ on EVs on NF-κB signaling in neurons in hippocampal slices of hyperammonemic rats.

We also found that hyperammonemia reduces TGFβ levels in hippocampus as well as the total content and membrane expression of TGFβ receptor 2. This should result in reduced function of pathways modulated by TGFβ and its receptors and may explain the reduction in the content of Smad7 and IkB in hyperammonemic rats.

A limitation of the study is that we have not assessed for how long the beneficial effects of MSCs-EVs remain in hyperammonemic rats. Future studies addressing this question would be useful to try to translate this treatment to clinical practice for patients with hepatic encephalopathy. Also, the study has been performed in a model of chronic hyperammonemia. This is a well-stablished model of minimal hepatic encephalopathy that reproduces mild cognitive and motor deficits found in patients. Further studies using animal models with liver failure may be also useful to better understand in detail the more appropriate conditions to translate the treatment to patients.

In summary (Fig. 12), this report shows that hyperammonemic rats show reduced levels of TGFβ and membrane expression of TGFβ receptors in hippocampus. This leads to activation of microglia to a pro-inflammatory phenotype and to reduced Smad7–IkB pathway, which induces nuclear translocation of NF-κB in neurons. Both microglia activation and NF-κB translocation induce an increase in IL-1β synthesis in microglia and neurons. The increased levels of IL-1β lead to enhanced activation of the IL-1 receptor which, in turn, alters membrane expression of AMPA and NMDA receptor subunits, leading to cognitive impairment.

EVs from MSCs injected i.v. to hyperammonemic rats reach the hippocampus and restore cognitive function. This improvement of cognitive function is mediated by TGFβ in the surface of EVs, which activates TGFβ receptors in microglia and neurons. This leads to a shift from a pro-inflammatory to a non-inflammatory state, which involves a reduced IL-1β production in microglia. Moreover, TGFβ also reduces nuclear translocation of NF-κB in neurons by normalizing the Smad7–IkB pathway. This also normalizes IL-1β production in neurons, reducing IL-1β in hippocampus to normal levels. This normalizes activation of IL-1 receptor and membrane expression of NR2B, GluA1 and GluA2 and, therefore, cognitive function.

## Conclusions

We show here that EVs from MSCs reduce neuroinflammation and restore cognitive function in rats with chronic hyperammonemia, a main contributor to MHE and clinical HE. The results reported support the idea that EVs from MSCs would also improve cognitive function in patients with liver cirrhosis and MHE or clinical HE. Currently, no specific treatments are available to reverse the neurological alterations in patients with MHE that affects more than 5 million people in USA [50] and many more millions around the world. Only the use of rifaximin, a non-absorbable antibiotic, has been approved for prevention of appearance of clinical HE episodes [7, 8, 60]. There is therefore a need for safe and efficient treatment of the neurological alterations on patients with MHE. The results presented in this report support that the use of EVs from MSCs may cover this need and improve cognitive function in patients with MHE or clinical HE.

## Data Availability

The data that support the findings of this study are available from the corresponding or first author, upon reasonable request.

## Abbreviations

Arg1: Arginase 1
CD9: Cluster of differentiation 9
Dil: 1,1′-Dioctadecyl-3,3,3′,3′-tetramethylindocarbocyanine perchlorate
EVs: Extracellular vesicles
GluA: Glutamate receptor
cGMP: Cyclic guanosine monophosphate
HA: Hyperammonemia
HE: Hepatic encephalopathy
Hsp70: Heat shock protein 70
Iba1: Ionized calcium binding adaptor molecule 1
IFNγ: Interferon gamma
IkB: Inhibitor of nuclear factor kappa B
IL-1β: Interleukin 1 beta
IL-4: Interleukin 4
IL-6: Interleukin 6
IL-10: Interleukin 10
MHE: Minimal hepatic encephalopathy
MSCs: Mesenchymal stem cells
NeuN: Neuronal nuclei
NF-κB: Nuclear factor kappa B
NOL: Novel object location
NOR: Novel object recognition
NR2B: N-Methyl-d-aspartate receptor 2
Smad7: Mothers against decapentaplegic homolog 7
TGFβ: Transforming growth factor
TGFβR: Transforming growth factor receptor
TNFα: Tumor necrosis factor alpha
TNFR1: Tumor necrosis factor alpha receptor
